# Mood dynamics in adolescents and young adults with and without a history of suicidal thoughts and actions: a network approach

**DOI:** 10.1186/s12888-026-08273-w

**Published:** 2026-06-11

**Authors:** Catharina Voss, Hanna Kische, Theresa M. Ollmann, Frank Rückert, Jana Hoyer, Katja Beesdo-Baum

**Affiliations:** 1 Behavioral Epidemiology, Institute of Clinical Psychology and Psychotherapy, TUD Dresden University of Technology, Chemnitzer Straße 46, D-01187 Dresden, Germany; 2 Obesity Day Unit, Städtisches Klinikum Institution, Dresden, Germany

**Keywords:** Network analysis, EMA, Suicidal behavior, Self-harm

## Abstract

**Background:**

Technological advances can be useful to overcome the mainly retrospective research in the field of suicidal behavior. Integrating established theories on suicidal behavior, namely the dynamic system theory and the network theory, the goal of the present study was to examine real life moment-to-moment mood networks between anhedonia, anxiety, depression, hopelessness, irritability, and stress in individuals with a history of suicidal thoughts only, suicidal actions, and no suicidal behavior.

**Methods:**

A history of suicidal thoughts (wish, ideation) and actions (plan, attempt) was assessed face-to-face using a standardized interview in a random community-based sample of individuals aged 14 to 21 years from Dresden, Germany (*N* = 1,180, response rate 21.7%). Mood states were examined using smartphone-based Ecological Momentary Assessment (EMA) on four consecutive days eight times a day. The analysis sample included *n* = 1,072 participants with available EMA data consisting of a suicidal thought group (*n* = 94), a suicidal action group (*n* = 76), and a no suicidal behavior group (*n* = 902). Using vector-autoregression analyses (R package mlVAR), contemporaneous and temporal networks were determined.

**Results:**

Descriptive results revealed quite similar contemporaneous networks in all groups, except for differences in some edge weights. Temporal networks differed in the overall density pointing towards stronger associations between mood states in the suicidal action group. Here, additional analyses revealed interaction effects by group in the associations between irritability and anhedonia, irritability and stress, anhedonia and hopelessness.

**Conclusions:**

Future research should consider these patterns in trying to explain why some people act on their suicidal thoughts.

**Supplementary information:**

The online version contains supplementary material available at 10.1186/s12888-026-08273-w.

## Background

Traditional research on predictors of suicidal behavior (SB) focuses mostly on demographic characteristics, mental disorders, and life events as risk factors for SB [1]. More recently, studies take advantage of modern technologies [2] to derive novel insights into more proximal predictors. In this context, studying real life moment-to-moment changes in mood could be an essential step [1, 3, 4]. Variances in mood networks [5–8] like pathways and density might allow a differentiation between individuals with suicidal thoughts only compared to those with suicidal actions (i.e. a plan or attempt). Suicide is a major public health concern around the world [9] – especially in adolescents and young adults [10]. Though non-fatal suicidal behaviors are much more common [11–14], with aggregated lifetime prevalence estimates of 18.0% for suicidal ideation, 9.9% for suicidal plan, and 6.0% for suicide attempt [14]. A recent study from the United States found even higher 12-month prevalence rates for suicide plans and attempts in 2021 [12].

Current theories for SB are mostly built upon diathesis-stress models and allow a theoretical differentiation between thoughts and actions based on the Ideation-to-Action framework [15, 16]. According to the Integrated Motivational-Volitional model of SB, individuals go through a motivational and volitional phase in the process from thoughts to actions [17]. In the motivational phase, suicidal ideation and intent develop based on feeling of defeat, humiliation, and entrapment in a stressful internal or external condition. In the volitional phase, steps towards suicidal actions are enhanced by factors like previous suicide attempts and impulsivity [17]. Current theories on cognition and emotion, e.g. the extended emotional regulation modal model [18], state that individuals with SB show dysregulation in the level of goal and action identification, e.g. higher level of self-awareness and representations of maladaptive regulation strategies [18] and thereby an increased decision-making bias for active responses to escape aversive states [19]. While those with helplessness show difficulties in action formation based on a general regulatory goal, those with a suicide attempt seem to act more on instant proximal goals and show a cognitive bias on immediate movements and sensations [18].

These frameworks describe internal states on a micro-level of psychopathology [20], meaning a continuous dynamic interplay between moment-to-moment experiences and behavioral patterns over time, which may determine the transition from a healthy to a non-healthy state. So far, internal states have been assessed predominately in a retrospective way in suicide research [3, 21]. The ecological-momentary assessment (EMA) method facilitates to capture momentary real life cognition, emotion, and behavior to get an ecological valid understanding [20, 22]. Research assessing these internal states and their dynamic interplay in real life in individuals with SB and thereby more proximal indicators for suicidal action is scarce [23, 24], but an important next step [25].

One study assessed defeat and entrapment using EMA in a small sample of healthy adults (*N* = 61) and showed temporal instability and variability of these constructs [26]. These results point to unknown underlying mechanisms leading to the experience of defeat and/or entrapment in daily life. Investigating more broadly different mood states in individuals with and without a history of SB appears useful to identify underlying mood dynamics. Here, the idea of the network approach plays a crucial role - mental disorders develop by the causal interplay between symptoms and their underlying moment-to-moment affect dynamics, and not a common cause [6, 8, 20, 27, 28].

The present study takes advantage of available EMA data from an epidemiological study among adolescents and young adults to explore pathways between varying mood states in youth with and without a history of SB using the network approach [5–7]. We differentiate those with thoughts only from those with actions according to the motivational and volitional phase of the Integrated Motivational-Volitional model of SB [17] and in accordance with a previous study indicating that wish to die and/or suicidal thoughts show a similar association with suicide attempt [29]. Based on the theoretical considerations described above, the following mood states were considered: anhedonia, anxiety, depression, hopelessness, irritability, and perceived stress. Anhedonia, depression, hopelessness and irritability are core symptoms of depressive disorders in adolescents and young adults [30] and were chosen on a moment-to-moment level as the concepts of defeat and entrapment were firstly discussed in the context of studying depressive disorders [31, 32]. As previous studies showed associations between defeat and entrapment with anxiety [31], anxiety on a moment-to-moment level was included. Finally, perceived stress was chosen to integrate an indicator of possible internal or external stress which could trigger the dynamic interplay or gets triggered by the basic mood states. The contemporaneous (same moment) and temporal (present time (t) predicted by the previous time point; *t*-1) networks were determined in three groups: history of suicidal thought (i.e. wish or ideation) only, history of suicidal action (i.e. plan or attempt), and no SB. We expected that 1) stronger moment-to-moment associations, previously described as “vicious cycles” [6, 33], between the included constructs should be observed in those with SB compared to those without illustrated by higher densities of the networks, and 2) higher inertia of irritability (the carry over effect of irritability from one moment to the next) as well as a stronger association between irritability and stress should occur in those with suicidal actions compared to those with suicidal thoughts only. The latter assumption is based on findings that irritability is a mood state which could drive approach behavior [34–36] and thereby could increase the possibility for behavior engagement (suicidal actions) due to higher and/or lasting stress over time [37] in the context of volitional factors [17]. Differences in the networks between the analyzed groups could emerge due to varying covariates. As SB is a symptom of a major depressive disorder [30] and often co-occurs with non-suicidal self-injurious behavior (NSSI) in adolescents and young adults [38], additional analyses were therefore conducted to determine the differences in the edges between groups including these covariates.

## Methods

### Study design

Data come from the baseline investigation of the Behavior and Mind Health (BeMIND) study, an epidemiological cohort study of adolescents and young adults from Dresden, Germany [39]. In 2015, a sex- and age-stratified sample of 14 to 21 year olds was randomly drawn from the population registry of the City of Dresden and *N* = 1,180 were assessed (response rate: 21.7%) between 11/2015 and 12/2016 after written invitation and a maximum of two reminder letters. Lack of time and lack of interest to participate were the most common named reasons for non-participation. Factors linked to higher probability of participation were higher education and concern for physical or mental health issues [39]. Assessments included a clinical-diagnostic interview, an experimental assessment approximately one week later, and an EMA part in real life as well as an online questionnaire assessment in between these two personal appointments. All participants and in minors also their legal guardians gave their written informed assent or consent. The BeMIND-study has been performed in accordance with the ethical standards laid down in the 1964 Declaration of Helsinki and its later amendments and the study protocol has been accepted by the ethics committee of the Technische Universität Dresden, Germany (EK381102014). Participants received 50 Euro as incentive for participation in the baseline assessment [39].

### Measures

#### Lifetime suicidal behavior

An updated research version [40] of the fully standardized computer-assisted Munich-Composite International Diagnostic Interview (DIA-X/M-CIDI [41]); was used to assess SB face-to-face by trained clinical interviewers (DIA-X-5; time range 1.5 to 8 hours). Within the depression section of the interview, four dichotomized items assessed wish to die (“Have you ever had the wish to die over a period of days or weeks?”), suicidal ideation (‘Have you ever thought about taking your life (i.e. attempt suicide) over a period of days or weeks?’), plan (if ideation: ‘Have you ever made a specific plan on how to kill yourself?’), and attempt (‘Have you ever attempted suicide?’) in all participants. If the participant affirmed one of these questions, a subsequent questionnaire was provided assessing the age at onset and last occurrence. There is no published psychometric data specifically on suicidality items for the CIDI [42], but reliability and validity of the instrument and the depressive disorder section have been established [40, 43], e.g. Cohen’s Kappa was 0.81 for any depressive disorder [40].

#### Momentary mood states

Via a self-developed study smartphone app, participants answered items regarding their daily life experiences during the four-day EMA period. Eight reminders to participate were individually set for each participant to optimally fit the assessment schedule to their everyday life (e.g., sleep times, school hours) for each of the four consecutive assessment days (two week-days and a weekend) resulting in a maximum of 32 assessments per participant. The assessment was divided into one morning, six midday and one evening assessment set with each containing between 203 and 248 items including filter/gate items to limit assessment burden. Participants were informed by trained staff about the EMA study procedures including the possibility to postpone each survey three times or skip an assessment as well as a training phase on the day before the first assessment.

In the present analyses, the following mood constructs were used with the timeframe: “Since the last beep …”: Anhedonia was assessed with a reverse-coded and self-developed item “I was able to enjoy things or activities” using a slider from 0 ‘no joy or pleasure’ to 100 ‘full joy or pleasure’. Anxiety was assessed with the mean of the 4-item Patient-Reported Outcomes Measurement Information System (PROMIS) Emotional Distress – Anxiety short-form scale [44] using a slider from 0 ‘never’, 50 ‘sometimes’, to 100 ‘always’. Depression and hopelessness were assessed each with an item of the 4-item PROMIS Emotional Distress – Depression short-form scale [44] using a slider from 0 ‘never’, 50 ‘sometimes’, to 100 ‘always’ (‘I felt depressed’; ‘I felt hopeless’). Irritability was assessed with the mean of the 5-item PROMIS Emotional Distress – Anger short-form scale [44] using a slider from 0 ‘never’, 50 ‘sometimes’, to 100 ‘always’. Stress was assessed with the self-developed item “I felt stressed” using a slider from 0 ‘never’ to 100 ‘always’. The scale was transformed from 0 to 100 to 0–10 for all six constructs. To reduce the daily burden for the participants, most constructs were assessed using single items and timeframes were adapted to the EMA setting [39], as no comprehensive itembanks existed for EMA [22, 45]. A pilot study was conducted testing usability. The use of single items is often discussed in regard to psychometric properties [45–47], but general psychometric measures don’t fit well with the EMA design [45, 48–50]. In the present study, descriptive EMA item information was calculated and weighted correlations between the aggregated mean of each of the six mood items and sum scores of retrospective standard symptom measures were conducted to get an idea of the criterion validity of the EMA items [46]. The within-person reliability for the four single items was calculated using the method described by Nezlek [51] and the computation described in Bonito, Ruppel and Keyton [52] using SAS 9.4. The reliability is determined by mixed effect models, using the maximum-likelihood method, with intercept as fixed effect and subject as well as occasions nested within subjects as random effects assuming an unstructured covariance structure. The reliability was moderate to substantial (anhedonia 0.88; stress 0.91; depression 0.72; hopelessness 0.70). Reliability of the used scales was also moderate (anxiety) to substantial (anger) [53].

#### Socio-demographic characteristics

At the beginning of the clinical interview, socio-demographic variables were assessed: sex, age, citizenship, current education status (high refers to at least A-Level vs. lower, middle or other), living arrangement, and subjective social class (lower, middle, upper).

#### Covariates for additional analyses

A history of any depressive disorder [40], i.e. major depressive or persistent depressive disorder (dysthymia), and non-suicidal self-injurious behavior [54, 55] was assessed during the interview.

### Statistical analysis

Stata software package 14.2 [56], R 3.6.1 [57] and SAS 9.4 [58] were used for analyses. Sample characteristics, post-hoc regression analyses as well as additional analyses were weighted to improve representativeness (sex and age) for the general population of the city of Dresden [39]. Frequencies (N/n) are reported unweighted. Adjusted Wald-Tests were used to test differences between socio-demographic variables and the three groups. Post-hoc tests were conducted using logistic regression analyses for significant results. The difference in compliance between the three groups was tested using linear regression analyses. Tests were done at the alpha-level 0.05 (two-sided).

#### Ecological momentary data

Only individuals with EMA data of 16 or more assessments (*t* ≥ 20: 96.5%; *t* ≥ 30: 56.53%) were included in the analyses (*N* = 1,072) to reduce the risk for a Nickell’s bias in the autoregressive effects [59, 60]. Overnight lags were removed by including an empty row. Similar, if assessments were more than 3 hours apart, an empty row was included too, whereby no associations were calculated between lags > three hours. An assessment less than 30 minutes apart from the previous one was deleted (20 observations). The overall mean (mean of each EMA construct) and variability (standard deviation) of each EMA with 95% CI were determined across the four assessment days. Linear regression analysis was used to test differences between the groups. For variability, analyses were adjusted for the person-mean as a higher person-mean automatically results in an increased standard deviation.

#### Network analyses

Unweighted two-step multilevel vector autoregressive (mlVAR) models [5] based on Gaussian graphical models were used. Nodes represent mood states and edges the estimated associations. Individual-specific and group-level estimates with average fixed and random effects were estimated based on restricted maximum likelihood estimation, a straightforward description of the exact time-series modelling can be found elsewhere [5, 61]. The networks were estimated using the estimator “orthogonal” (estimating non-correlated random effects in node-wise estimation) due to six nodes [5]. Two networks were conducted: 1) contemporaneous network, an undirected within-subject model illustrating cross-sectional associations of the mood states after taking temporal effects into account based on partial correlations; 2) temporal network, a directed within-subject network model illustrating the degree to which a current mood state at time point t can be predicted from itself and all other mood states at the previous time point (*t*-1). In the current study, time *t*-1 and time t refer to two consecutive beeps within the same day (30 minutes to 3 hours apart), whereby mood states were assessed with the timeframe “since the last beep”. This approach assumes stationary of the mean and the moment-to-moment associations of the mood states over time [5, 62], i.e. that the time series is constant over time with a unit root [59]. Current findings regarding key assumptions of mlVAR models are well described by Jordan and colleagues [59]. The assumption of normality of the residual distributions was visually checked for each analyses showing some violations. Two assumptions were confirmed: 1) approximately equal time intervals between assessments referring to the described study protocol, 2) stationarity of the data indicating that the mean and variance of the time series data remains. To examine stationarity, i.e. an unit root, Fisher-type unit-root tests based on augmented Dickey-Fuller test [63] were run for each mood state in the total sample indicating stationary in at least one panel (*N* = 1,072, mean number of assessments 26.73 to 26.96, *p* < 0.001). To test every participant individually for each mood state, the procedure was rerun adjusting for multiple tests. Results indicate that more than 98% of the data can be assumed stationary.

Only significant effects at *p* < 0.05 were visualized in the temporal networks as well as the conservative “and”-rule was used for the contemporaneous network indicating that both *p*-values need to be significant [5]. All network analyses were run in R 3.6.1 using the packages qgraph [64] and mlvar [5], the used R code can be found in Additional file A4.

Different network indicators were calculated: density as the average network connectivity [65] was determined by the mean of all (significant) edge weights (contemporaneous network: mean of partial correlations; temporal network: mean of fixed effects). The density based on all internode connections (mean of all (significant) edges between different nodes) and (significant) self-loops were determined [65]. Using the comment centrality of the R package qgraph [64], the indegree and outdegree were determined in the temporal networks and the strength in the contemporaneous networks. In/Outdegree was calculated by summing the fixed effect estimates from the regression models for the different directed edges without auto-correlations.

Like most previous studies, visual comparison of the estimated parameters was used to compare networks [61]. No general indicator exists to test the overall differences between several time-series networks [33, 61, 66]. Therefore, edge weights of >0.05 were described in the results. Differences between the three groups in all edge estimates were conducted and differences of >0.07 (contemporaneous) and >0.09 (temporal) were described in the result section as well as tested again in additional analyses. In network analyses, no consensus regarding effect sizes or minimal clinical-important differences exists, so the values of >0.05 (mean of all fixed effects in the temporal model using the total analyzed sample, *n* = 1,072), >0.09 (two times the mean in the temporal network), >0.07 (two times the standard deviation (0.04) or higher in the contemporaneous network) were chosen based on the current data. Based on recent research [61], additional analyses were conducted using a nonparametric permutation test (see Additional file A3).

### Additional analyses

Information regarding the additional analyses can be found in the Additional files A2 and A3.

## Results

### Sample characteristics

Of the *N* = 1,180 BeMIND participants, *n* = 1,158 (98.10%) took part in the EMA. Of those, *n* = 1,072 (90.85%, 51.20% male, mean age 17.93, 97.22% German citizenship, 77.98% high education status, 17.54% lower and 60.57% middle subjective social class) had sufficient assessment to be included in the present analyses. The analyzed sample reported significantly more often a higher education status (OR = 2.21, 95% CI 1.42–3.45, *p* < 0.001) and a history of SB (OR = 3.60, 95% CI 1.41–9.17, *p* = 0.007) than the non-analyzed group (*n* = 108), no differences were found for sex, age group, citizenship, living arrangement, subjective social class, NSSI, and any depressive disorder.

Table 1 depicts the sample characteristics (socio-demographics, covariates, EMA-study variables). Three groups were defined based on lifetime SB: suicidal thought group (*n* = 94), suicidal action group (*n* = 76), no SB group (*n* = 902); *n* = 101 with no SB, *n* = 3 with suicidal thoughts, and *n* = 4 with suicidal actions were not included in the analysis due to less than 16 EMA. The suicidal action group significantly differed from the no SB group by a lower current education status (OR = 0.47, 95% CI 0.28–0.80, *p* = 0.005), and the suicidal action compared to the suicidal thought only group was characterized by a higher proportion of females (OR = 2.08, 95% CI 1.01–4.30, *p* = 0.047) and NSSI (OR = 2.07, 95% CI 1.06–4.06, *p* = 0.034). EMA compliance did not differ by group (Table 1).

**Table 1:** Sample characteristics based on the socio-demographic, covariate and EMA variables

	Suicidal thoughts (*N* = 94)	Suicidal actions (*N* = 76)	No suicidal behavior (*N* = 902)	Adjusted Wald-Test χ2, *p*
	*n* (%w)	*n* (%w)	*n* (%w)	
**Socio-demographic variables**				
Sex				5.56, *p* = 0.004
female	60 (50.85)	59 (68.31)	511 (47.03)	
male	34 (49.15)	17 (31.69)	391 (52.97)	
Age Group				1.18, *p* = 0.313
14–15	26 (18.53)	15 (12.81)	273 (21.98)	
16–17	24 (19.86)	17 (17.98)	224 (20.02)	
18–19	22 (25.21)	26 (34.99)	210 (25.66)	
20–21	22 (36.40)	18 (34.21)	195 (32.33)	
Citizenship				0.84, *p* = 0.431
German	89 (93.58)	74 (96.95)	883 (97.61)	
non-German	5 (6.42)	2 (3.05)	19 (2.39)	
Current education status				3.38, *p* = 0.034
low, middle, other	23 (25.75)	30 (35.38)	204 (20.57)	
high	71 (74.25)	46 (64.62)	698 (79.43)	
Living arrangement				0.60, *p* = 0.728
with parents	72 (66.76)	54 (65.30)	667 (63.51)	
alone	10 (15.27)	9 (15.35)	84 (12.49)	
with partner	3 (3.31)	5 (6.78)	38 (5.49)	
other	9 (14.65)	8 (12.58)	113 (18.40)	
Subjective Social Class				1.64, *p* = 0.161
lower	16 (21.73)	17 (24.03)	123 (16.59)	
middle	52 (55.23)	48 (63.25)	548 (60.89)	
upper	24 (23.04)	11 (12.72)	213 (22.52)	
**Covariates**				
Lifetime NSSI	39 (39.35)	49 (57.33)	78 (7.75)	36.92, *p* < 0.001
Lifetime depressive disorder	44 (47.68)	49 (58.68)	86 (10.23)	37.11, *p* < 0.001
**EMA characteristics**				Kruskal-Wallist Test χ2, p
Total number of assessments	2,812	2,216	26,974	n.a.
Completed assessments	2,515	1,971	24,171	n.a.
Compliance (%), mean [95% CI]	84.48 [81.72–87.24]	82.89 [79.55–86.23]	84.83 [83.91–85.75]	2.440, *p* = 0.295

*Note.* Frequencies (N/n) are reported unweighted and percentages as well as mean scores were calculated using sample weights. Adjusted Wald- and Kruskal-Wallist Tests at *p* < 0.05 were used to test differences between socio-demographics, covariates and EMA characteristics and the three groups. Abbreviations: NSSI: non-suicidal self-injurious behavior; n.a. not applicable

### Mood state characteristics

Additional file A1 Table A1.1 depicts the descriptive information including the overall mean and variability of each of the six mood states across the four assessment days. Those with a history of suicidal thoughts and actions showed significantly higher mean estimates than the no SB group. No significant group differences were found regarding variability. Additional file A1 Table A1.2 shows the weighted correlations of the six mood states with standard retrospective symptom scales allowing us to get an idea of the concurrent validity. Overall, correlations of EMA constructs were mostly highest with corresponding retrospective scale scores, particularly in the corresponding time frame, suggesting valid EMA.

### Network analysis

**Fig. 1:**
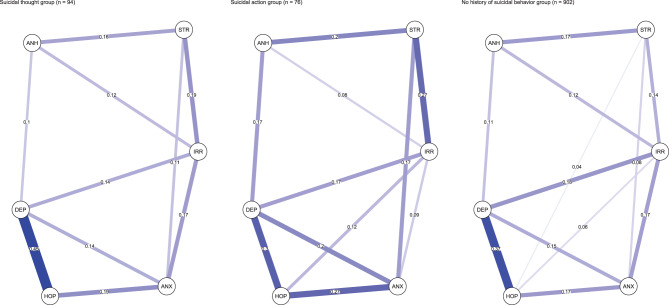
Contemporaneous networks for each group showing significant paths. Note. the Fruchterman-Reingold algorithm was used to visualize the network using the packages qgraph [64] in R version 3.6.1. The maximum strength of the networks was set equal for all temporal networks (0.25). Blue edges indicate positive associations, red edges indicate negative associations. Only significant effects at *p* < 0.05 were visualized using the conservative “and”-rule was used for the contemporaneous network indicating that both *p*-values need to be significant [5]. Abbreviations: ANH: anhedonia, ANX: anxiety, DEP: depression, HOP: hopelessness, IRR: irritability, STR: stress

**Fig. 2:**
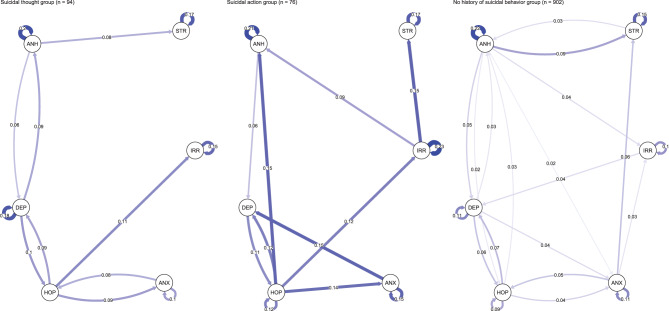
Temporal networks for each group showing significant paths. Note. The Fruchterman-Reingold algorithm was used to visualize the network using the packages qgraph [64] in R version 3.6.1. The maximum strength of the networks was set equal for all contemporaneous networks (0.45). Blue edges indicate positive associations, red edges indicate negative associations. Inertia as the carry over effect from one moment to the next is represented by self-loops based on autoregressive effects. Only significant effects at *p* < 0.05 were visualized in the temporal networks [5]. Abbreviations: ANH: anhedonia, ANX: anxiety, DEP: depression, HOP: hopelessness, IRR: irritability, STR: stress

The results of the network analyses are shown in Fig. 1 (contemporaneous network) and Fig. 2 (temporal network). The contemporaneous network of the suicidal thought group showed 10 edges above an edge weight of 0.05 and the suicidal action and the no SB group 11 edges each. The temporal network of the suicidal thought group showed eight significant edges and five significant self-loops/auto-correlations, the suicidal action group nine significant edges and five significant self-loops, and the no SB group four edges and six self-loops above an edge weight of 0.05.

#### Contemporaneous network

The contemporaneous networks were similar regarding significant positive associated edges in all groups. Irritability was the only construct significantly associated with all other mood states, except for no significant edge between hopelessness and irritability in the suicidal thought group. For example, a significant association can be found between irritability and stress indicating that in the presence of irritability stress occurs more likely or vice-versa. Differences in edge weights of >0.07 between the groups were found for the association between depression and hopelessness (Additional file A1 Table A1.3).

#### Temporal network

In accordance with the ideation-to-action framework, the three groups showed some different significant paths in their temporal networks. In all three groups, anhedonia significantly predicted depression in the next time point and a significant self-loop of anhedonia, anxiety, and stress could be observed. In the suicidal thought only group with individuals experiencing the motivational phase only in the past, a significant self-loop also occurred for irritability and depression. Further, anhedonia predicted stress; hopelessness predicted irritability, anxiety, and depression; anxiety and depression both predicted hopelessness; and depression predicted anhedonia. In the suicidal action group with individuals experiencing the motivational and volitional phase in the past, a significant self-loop for irritability and hopelessness could be observed. Further, irritability predicted stress and anhedonia; hopelessness predicted irritability, anhedonia, anxiety and depression; anxiety predicted depression and depression predicted hopelessness. In the no SB group, anhedonia predicted stress, irritability, and hopelessness; anxiety predicted anhedonia, hopelessness, and irritability with an edge weight above 0.05. Differences in the edge weights of >0.09 between the groups can be found in Additional file A1 Table A1.4. Higher estimates of the following edges were found in the action compared to the thought group: anxiety predicting depression; irritability predicting stress and anhedonia; hopelessness predicting anhedonia; but lower estimates for depression predicting depression (self-loop). No difference in the edge weights of >0.09 were found between the thought and no SB group. Higher estimates were found in the suicidal action compared to the no SB group regarding anxiety predicting depression, irritability predicting stress, anhedonia and irritability (self-loop), and hopelessness predicting anxiety, irritability, and anhedonia. According to the output of the mlVAR estimation in R, *n* = 42 in the suicidal thought group, *n* = 41 in the suicidal action group, *n* = 387 in the no SB group had less than 20 assessment lags, which might result in Nickell’s bias.

### Network indicators

The overall density for the contemporaneous and temporal networks was highest in the suicidal action (0.19, 0.14), compared to the suicidal thought (0.18, 0.12) and no SB group (0.15, 0.07), respectively (Additional file A1 Table A1.5). Similar results were found for the internode and self-loop density in the temporal networks. Network indicators for each mood state and group defined by the strength for the contemporaneous networks and outdegree and indegree for the temporal networks can be found in Additional file A1 Table A1.6.

### Additional analyses

For the results of the additional analyses, see Additional file A2. Shortly, an interaction effect by group was found in the associations between irritability and anhedonia, irritability and stress, anhedonia and hopelessness. Using the nonparametric permutation test (see Additional file A3), significant stronger associations were found in the suicidal action group compared to the suicidal thought group in the aforementioned associations as well as between anxiety and depression. The same results were found comparing the no suicidal behavior and the suicidal action group, and additionally a significant self-loop of irritability emerged.

## Discussion

For the first time, the present study showed varying mood networks in real life in individuals with a history of suicidal behavior, differentiating thoughts only from actions, and compared to those without SB. Using an EMA approach in an epidemiological study of adolescents and young adults, findings illustrated that a history of SB was associated with higher experience of all six mood states (anhedonia, anxiety, depression, hopelessness, irritability, and stress) in daily life. Examining the contemporaneous and temporal networks in the three groups based on conditional dependencies, a higher density was found in the SB groups compared to the no SB group predominantly in the temporal network structures. Especially the density of the associations in the temporal network of the suicidal action group was two times the density of the no SB group indicating higher associations between the measured constructs in the action group. The present results showed that general population youth with a history of SB might be affected in their mood dynamics in daily life with a higher burden in those with past suicidal actions.

By nature, network analyses point out various associations between the included constructs. The following discussion will focus on the a priori assumptions.

### Mean differences by groups

The present results showed lower mean scores in all constructs in those without a history of SB. These findings are in line with previous EMA studies elucidating that individuals with current or a history of SB have impaired negative affect [23, 24] and positive affect (higher anhedonia) in daily life [67, 68]. These results might be an explanation for the findings of longitudinal studies pointing to long-term negative health consequences in adulthood in individuals with SB during adolescence [69, 70]. In contrast to previous findings, no differences in the variability of mood states were found [24, 71, 72].

### Density differences

Regarding differences in the density of the networks, previous studies showed that higher density was associated with more psychopathology [33, 73]. Some studies found no or reversed significant association with psychopathology [73]. In the present study, the highest density was found in the suicidal action group in the temporal network pointing to higher associations between the included key symptoms of depression, anxiety, and stress. This is in line with retrospective studies showing that SB is associated with the number of disorders, including anxiety and depression [74]. A higher number of disorders is accompanied by more symptoms and more mood disturbances on the micro-level according to the Complex Dynamic System Theory [20]. Alternatively, a higher density could also be a marker of higher emotional reactivity in general rather than psychopathology, underlying the extended emotional regulation modal mode [20]. Both interpretations might explain the negative long-term health consequences in individuals with SB. Due to previous varying results, differences in density between the groups could be an artefact of measurement error.

### Edge differences

Looking at the key differences in the edges between the three groups, the following were validated in the additional analyses showing a significant interaction between suicidal action group and the predictor compared to the no SB group: a) irritability predicting stress, b) irritability predicting anhedonia, c) hopelessness predicting anhedonia.

Firstly, the assumption was confirmed that in the suicidal action group irritability is more strongly connected with itself as well as with stress at the same timepoint and from one moment to the next. Secondly, irritability was also associated with more anhedonia at the next time point in those with a history of suicidal actions. These results indicate that if a person with past suicidal actions experiences irritability described as the proneness to anger, it may last longer and result in higher perceived stress but also loss of pleasure or joy. The predictive role of increased irritability during childhood for later SB was suggested in several studies [34–36], but EMA studies on the association between anger and SB show inconsistent results [23, 24]. The present study points to an important role that irritability may play in the formation of affect dysregulation. Brotman and colleagues [34] formulated a pathophysiological model of irritability in youth, where irritability is defined by an aberrant reward processing and an aberrant threat processing. Shortly, the aberrant reward processing refers to reward receipt and reward omission, and it is reflected by higher frustrative non-reward. Here, aberrant threat processing comprises an increased orienting to threat and a tendency to interpret the behavior of others as well as ambiguous situations as threat. In the context of threat, the individual experiences anger when it seems impossible to leave the situation [34]. Both processes interact with each other, so that in the context of experiencing threat, the reward sensitivity is reduced and thereby increases frustration due to a reduced experience of reward. This constellation results in the experience of stress to fight, flight or freeze to survive the unbearable situation [75]. This might result in the arrested flight mode described by Gilbert and Allan [32] and the experience of defeat and entrapment. Research regarding acute stress responses in individuals with SB showed, for example maladaptive responses in the autonomic nervous system and the hypothalamic-pituitary-adrenal axis [37]. Both systems are responsible for the activation and regulation of the stress response, which might be altered in those with SB, leading to a stress cascade as a proximal process before SB [37]. The higher arousal/stress in perceived stressful situations, the decreased sensitivity for reward and attentional bias to threat might partly explain the dysregulation in the level of goal and action identification in individuals with SB [18, 76] and especially the immediate urge to act on proximal goals due to higher motor activity [34]. This is an interesting moment to capture in more detail in future studies, e.g., by studying cognitive, behavioral, and physiological features like heart rate variability, cortisol, so indicators of imminent stress.

Irritability was not just associated with stress at the same time point in those with suicidal actions, moreover irritability was associated with stress 30 minutes to 3 hours later pointing to a dysfunctional process keeping the focus on the blocked reward or threat and increasing the motor activity so that the individual perceives stress.

In addition, irritability was associated with anhedonia at the next time point. Anhedonia was mostly positively associated with SB including suicidal actions in retrospective studies, though there were inconsistent findings too [77, 78]. An association between anhedonia and reward-processing was verified in several studies, but the exact mechanism is unknown [79]. As anhedonia is a symptom of several mental disorders, it is often discussed as a transdiagnostic marker for psychopathology and therefore can be understood as a construct of the Positive Valence System in the Research Domain Criteria (RDoC) framework due to its relation with Approach Motivation, Initial Responsiveness to Reward Attainment, and Reward Learning [80]. Here research indicated that individuals with SB show a blunted neural response to rewards and value rewards less [81]. As behavior activation is low during anhedonia, the experience of reward is reduced, but research showed that activation treatment approaches could modify the functioning of reward-related networks in the brain [80]. It seems contradictory that on the one hand anhedonia is correlated with less behavioral engagement while on the other hand is linked to increased risk of suicidal action. Here, the experience of defeat might play a role as it seems to be associated with anhedonia [31]. It could be speculated that persisting high anhedonia is linked to lower experience of positive reward and thus to a perception of defeat and increased stress. There is evidence that anhedonia is associated with maladaptive stress processing [82]. As shown in the mood networks, anhedonia is associated with stress at the same time point as well as from one time point to the next.

Overall, the experience of irritability in daily life is more strongly associated with perceived stress and loss of interest over time in those with a history of suicidal actions compared to those without.

In addition, anhedonia was associated with hopelessness at the previous time point in those with a history of suicidal actions compared to those without. Hopelessness showed an association with suicidal ideation in previous EMA studies [23, 24] and is a known risk factor for SB in prospective studies [1]. However, results regarding the prediction on the ideation-to-action transition are inconsistent [15]. Further studies are necessary to explain under which conditions hopelessness determines a transition to actions.

### Network centrality: strength

The validity of centrality measures in network analyses and their interpretability is discussed in several papers [59, 83, 84], with no final recommendation yet [7]. Predominantly, there are no recommended tests for testing the stability of centrality measures in temporal networks [59]. The results of the strength indicators underpin the above-mentioned result that the three groups differ in the assessed mood states. Overall, the contemporaneous indicators illustrate that except for anhedonia, both SB groups had higher strengths in all mood states compared to the no SB group. These results point to stronger associations in that the mood states affect each other more at the same time point. The strength of anhedonia was lowest in the suicidal thought group, followed by the no SB and finally suicidal action group. In the temporal networks, the irritability and hopelessness outdegree strength were descriptively higher in suicidal action group to both other groups, while for anhedonia outdegree strength was smaller and indegree strength higher than in both other groups. The depression indegree strength was doubled in the suicidal action group compared to both other groups. The results extend the information about the above-mentioned roles of irritability, anhedonia, and hopelessness. Interestingly, although anhedonia is associated with irritability and hopelessness from one moment to the next, its outdegree strength is low in those with suicidal actions. Here, anhedonia is only associated with stress, depression and irritability at the same time point and depression at the next time point. Further studies should assess the imminent emotional, cognitive, and behavioral correlates in the daily life experience of anhedonia. Beyond the centrality measure of strength, other measures like predictability [85] and expected influence [86] could be used to find central nodes in further studies.

As stated above, it remains unclear whether and how network results might inform clinical practice. Based on the present results, it could be hypnotized that individuals with a history of suicidal behavior differ in their daily experience of irritability, hopelessness, and anhedonia, and experimental studies could test the effects if these were to targeted, address either directly or indirectly. Regarding available treatments or interventions addressing irritability, only a few are available for adolescents and young adults [34]. Some interventions exist for reducing anhedonia [87], including behavioral activation approaches [88]. Besides these approaches, interventions focusing directly on suicidal actions like the Attempted Suicide Short Intervention Program (ASSIP [89]); seems to be effective in targeting the suicide mode state in general [90]. Results of interventional studies could provide insights into causal relationships in mood networks. Overall, effective prevention and intervention strategies are strongly needed [91, 92].

### Limitations

Although especially temporal networks induce the impression of causality, this is not necessarily the case [93, 94] and they are based on conditional dependencies of the included constructs, so the present findings should be interpreted with caution and need further empirical support based on experimental designs, e.g. in clinical samples. Regarding node validity, mood states were chosen based on theoretical considerations fulfilling the criteria of separately identifiable and independently manipulable constructs [83]. Still, mood states are all part of the depression symptom spectrum and moderately correlated. Further, mood states were mostly assessed with single items and questionnaires developed for retrospective studies, so the validity in EMA is not fully understood and needs to be thought of [83]. Though, single items could be appropriate [47]. No common psychometric guidelines exist for EMA so far, an useful tool for studying EMA items was suggested by Siepe and colleagues [48]. The EMA design tries to capture real life fluctuation, e.g. in a mood state, making it difficult to differentiate and quantify the measurement error, therefore the reliability of the used measures is important - especially when using single items [45, 49, 95]. The present single items and scales showed moderate to substantial reliability [51], so measurement error based on item or scale reliability could influence the associations in the shown networks by over- or underestimating the effects [95]. The mood states were associated with standard symptom measures in the present study, pointing to validity of the assessed constructs. The distribution of most mood states was skewed to the left and variance of included mood states was in most variables below the used cut-off to avoid a floor effect by Kuranova and colleagues [33] keeping items with a within-person standard deviation of around 1.0. Other aspects of measurement designs seem to play a role in the magnitude of measurement error in time-series data [49, 50]. For example, it is unclear how the phrasing „since the last beep” influenced the reliability of the participants answers, so further studies are necessary comparing reliability effects of different timeframes including phrases like “at the moment …” [45, 96]. The mlVAR approach is based on some critical assumptions, which could easily be violated [5]. For example, the assumption of stationary might not be applicable, illustrating the need for other approaches to capture breaks and changes [50, 83, 94] without losing information [50]. There are some recent ideas of different time-series modelling to overcome these issues [62, 85, 97]. Beyond modelling techniques, it seems necessary to study the network structure again in a clinical sample with higher symptomatology. Further, different study designs regarding time intervals between assessments (minutes and hours) would be interesting to get an understanding of the lasting impact of the vicious cycles, i.e. the association between the mood states from moment-to-moment.

Another limitation of the present study is the definition of the analyzed groups. Due to a small number of participants with current SB, those with a history were included in the present analyses, so that the question “When is risk most acute?” [25] cannot be answered with the present findings. Further, individuals with wish to die and suicidal ideation were combined in the suicidal thought group and those with a plan or attempt in the suicidal action group. However, individuals endorsing these different types of SB on the ideation-to-action path might differ in their mood networks. Still, the categorization was based on the two phases of the Integrated Motivational-Volitional model of SB [17] and can be understand as a first step to reveal mixed mood dynamics. While the inclusion of depressive disorders and NSSI as covariates is a strength in our study, other potential confounders (e.g., impulsivity, substance use, protective factors) were not considered. As these (and other) factors could influence mood dynamics and suicidal behavior, one needs to keep the potential role of other factors in mind. Potential bias needs to be addressed given the limited overall response rate in our study, with higher education and prior treatment for physical and mental health conditions being linked to participation [39], and given some differences in analyzed vs. not analyzed subjects, with higher education and history of SB being linked to data availability. Keeping in mind that no study ever can achieve complete representativeness and given that larger bias is more likely when estimating marginal parameters (such as prevalences/incidences) than when estimating associations or effects [98] and bias might be further smaller when moderators of associations are investigated, our results are expected to be largely transferable to the somewhat better educated adolescent and young adult population of Dresden with higher health concern. With regard to generalizability to other populations within Germany or internationally, urbanicity and a low proportion of non-German citizenship and migration background of our Dresden sample should be noted [39]; thus our findings apply to a rather homogenous population.

The EMA compliance was higher than in previous studies and above the recommended 80% [25, 59]. But still some participants had less than 20 assessment lags which increases the risk for a bias – especially in temporal network estimation [99]. For example, a downward bias in the estimations of auto-regressive effects was found [99], known as Nickell’s bias [59, 60]. At the current stage, we could only point to this estimation bias and the need for further studies testing the present hypotheses with different tools. This is also the case in the multi-level regression analyses used as additional analysis tool. Here, centering was used as well which could have led to a bias, especially in auto-regressive estimations [99]. The results of the additional analyses do not falsify the network structure if the results are not significant for specific edges, because the analyses included only one mood predictor instead of all mood states.

Current network structures might not be applicable to all individuals with a history of SB [100], so that personalized network analyses need to be conducted in the future [25, 59, 101]. This research could inform clinicians about the most efficient treatment target. There is a lack of clear guidelines which target to choose leading to very different approaches so far [102]. More important, sufficient sampling is mandatory, and methodological considerations are required [94].

Network models based on time-series data are a novel approach in psychological research. Therefore, statistical considerations vary largely and change quickly [83]. Centrality measures need to be carefully interpreted and indicators for comparing time-series networks are still scarce and need further considerations to allow direct comparison [61, 66]. Before using these network structures in clinical practice, a replication is necessary using different approaches, e.g. Bayesian multivariate estimation [5] or adapted VAR modeling [62, 83, 97, 103].

## Conclusions

The present study can be one step to further generate hypotheses in studying proximal antecedents for suicidal behavior and especially for the Ideation-to-Action transition. For example: In which moments does the network shift to a state that increases the risk for acute suicidal behavior? In this realm: How do (internal/external) contextual factors trigger these shifts and can be captured? Future studies could analyze the included mood dynamics in the context of the dynamic system theory [20] by studying early warning signs [101]. The crucial question to be resolved is: How do individuals most at risk can be identified in an instant and valid way?

## Electronic supplementary material

Below is the link to the electronic supplementary material.

Supplementary Material 1

Supplementary Material 2

Supplementary Material 3

Supplementary Material 4

## Data Availability

The datasets used and/or analysed during the current study are available from the corresponding author on reasonable request.

## Abbreviations

ASSIP: Attempted Suicide Short Intervention Program
BeMIND: Behavior and Mind Health study
DIA-X/M-CIDI: Fully standardized computer-assisted Munich-Composite International Diagnostic Interview
EMA: Ecological Momentary Assessment
mlVAR: Multilevel vector autoregressive model
NSSI: Non-suicidal self-injurious behavior
OR: Odds ratio
PROMIS: Patient-Reported Outcomes Measurement Information System
RDoC: Research Domain Criteria
SB: Suicidal behavior
