# Supplementary material for: Mood dynamics in adolescents and young adults with and without a history of suicidal thoughts and actions: a network approach
Source: BMC Psychiatry. 2026 Jun 11;26:460. doi: 10.1186/s12888-026-08273-w (PMC13262410; doi:10.1186/s12888-026-08273-w)
Supplement: Supplementary file 1 — Supplementary Material 1 [file 12888_2026_8273_MOESM1_ESM.docx]

**Additional File 1**

Voss, C., Kische, H., Ollmann, T. M., Rückert, F., Hoyer, J., Beesdo-Baum, K. Mood Dynamics in Adolescents and Young Adults with and without a History of Suicidal Thoughts and Actions: A Network Approach

**Corresponding author:**

Prof. Katja Beesdo-Baum, PhD

TUD Dresden University of Technology

Institute of Clinical Psychology and Psychotherapy, Behavioral Epidemiology

Chemnitzer Strasse 46

D-01187 Dresden, Germany

Email: katja.beesdo-baum@tu-dresden.de

# Additional File 1 – Further Tables

**Table A1.1**

*Overall mean, variability, and group differences of the six mood states during EMA*

|  |  | Mean *[95% CI]* | ß | *[95% CI]* | *p* |  | SD *[95% CI]* | ß | *[95% CI]* | *p* |
| --- | --- | --- | --- | --- | --- | --- | --- | --- | --- | --- |
| Anhedonia |  |  |  |  |  |  |  |  |  |  |
| ST group |  | 3.60 [3.22-3.99] | Ref. |  |  |  | 1.99 [1.82-2.15] | Ref. |  |  |
| SA group |  | 3.52 [3.13-3.91] | -0.08 | [-0.62;0.46] | .769 |  | 2.08 [1.89-2.27] | 0.11 | [-0.13;0.35] | .379 |
| No SB group |  | 3.18 [3.07-3.29] | -0.42 | [-0.82;-0.03] | .036 |  | 1.89 [1.84-1.95] | -0.01 | [-0.17;0.15] | .904 |
| Anxiety |  |  |  |  |  |  |  |  |  |  |
| ST group |  | 0.64 [0.51-0.76] | Ref. |  |  |  | 0.63 [0.52-0.73] | Ref. |  |  |
| SA group |  | 0.69 [0.48-0.90] | 0.05 | [-0.19;0.29] | .668 |  | 0.69 [0.55-0.83] | 0.03 | [-0.09;0.16] | .581 |
| No SB group |  | 0.42 [0.38-0.45] | -0.22 | [-0.35;-0.09] | .001 |  | 0.44 [0.41-0.47] | -0.07 | [-0.14;0.00] | .053 |
| Depression |  |  |  |  |  |  |  |  |  |  |
| ST group |  | 1.03 [0.79-1.28] | Ref. |  |  |  | 1.21 [1.04-1.38] | Ref. |  |  |
| SA group |  | 1.25 [0.89-1.60] | 0.21 | [-0.21;0.63] | .318 |  | 1.32 [1.08-1.56] | -0.02 | [-0.22;0.18] | .829 |
| No SB group |  | 0.63 [0.57-0.69] | -0.40 | [-0.65;-0.16] | .001 |  | 0.85 [0.79-0.90] | -0.11 | [-0.23;0.01] | .076 |
| Hopelessness |  |  |  |  |  |  |  |  |  |  |
| ST group |  | 0.71 [0.54-0.88] | Ref. |  |  |  | 0.82 [0.66-0.98] | Ref. |  |  |
| SA group |  | 0.79 [0.49-1.09] | 0.08 | [-0.25;0.42] | .625 |  | 0.98 [0.76-1.20] | 0.11 | [-0.06;0.27] | .207 |
| No SB group |  | 0.44 [0.39-0.49] | -0.27 | [-0.45;-0.10 | .002 |  | 0.56 [0.52-0.61] | -0.08 | [-0.19;0.02] | .131 |
| Irritability |  |  |  |  |  |  |  |  |  |  |
| ST group |  | 0.77 [0.61-0.93] | Ref. |  |  |  | 0.81 [0.68-0.95] | Ref. |  |  |
| SA group |  | 0.81 [0.63-0.98] | 0.04 | [-0.20;0.27] | .746 |  | 0.90 [0.72-1.08] | 0.07 | [-0.09;0.23] | .403 |
| No SB group |  | 0.57 [0.52-0.62] | -0.20 | [-0.36;-0.04] | .017 |  | 0.64 [0.60-0.67] | -0.07 | [-0.17;0.03] | .180 |
| Stress |  |  |  |  |  |  |  |  |  |  |
| ST group |  | 2.26 [1.96-2.56] | Ref. |  |  |  | 1.93 [1.78-2.07] | Ref. |  |  |
| SA group |  | 2.27 [1.93-2.61] | 0.01 | [-0.44;0.45] | .981 |  | 2.03 [1.82-2.24] | 0.11 | [-0.10;0.31] | .319 |
| No SB group |  | 1.87 [1.78-1.95] | -0.39 | [-0.70;-0.09] | .012 |  | 1.78 [1.73-1.83] | -0.02 | [-0.14;0.11] | .810 |

*Note.* The range of the six mood states was from 0 to 10. Differences in the mean and variability were tested using weighted linear regression analyses and the suicidal thought group as the reference category. Analyses regarding the variability were adjusted for the person-mean. Abbreviations: EMA = ecological momentary assessment; No SB = no suicidal behavior group; Ref. = reference category; SA = suicidal action group; SD = standard deviation (variability); ST = suicidal though group.

**Table A1.2**

*Correlation of ecological momentary assessment (EMA) with retrospective information*

|  | Anhedonia | Anxiety | Depression | Hopelessness | Irritability | Stress |
| --- | --- | --- | --- | --- | --- | --- |
| **Before EMA** |  |  |  |  |  |  |
| PHQ-9 total | 0.27 | 0.28 | 0.40 | 0.32 | 0.25 | 0.31 |
| PHQ-9 item 1 anhedonia | 0.23 | 0.14 | 0.25 | 0.20 | 0.15 | 0.22 |
| PHQ-9 item 9 suicidality | 0.16 | 0.16 | 0.25 | 0.21 | 0.10 | 0.09 |
| Cross-D | 0.21 | 0.37 | 0.42 | 0.36 | 0.26 | 0.29 |
| ARI | 0.20 | 0.18 | 0.18 | 0.18 | 0.28 | 0.22 |
| PSS-4 | 0.37 | 0.39 | 0.46 | 0.42 | 0.36 | 0.40 |
| ASRM | -0.10 | 0.05 | 0.02 | -0.01 | 0.04 | 0.01 |
| LOT-R | -0.36 | -0.31 | -0.40 | -0.38 | -0.31 | -0.29 |
| OSLO-3 | -0.28 | -0.17 | -0.22 | -0.23 | -0.18 | -0.16 |
| **During EMA** |  |  |  |  |  |  |
| DSM-5 Level 1 total | 0.30 | 0.47 | 0.51 | 0.45 | 0.42 | 0.45 |
| DSM-5 Level 1 depression | 0.38 | 0.40 | 0.54 | 0.47 | 0.36 | 0.38 |
| DSM-5 Level 1 anger | 0.24 | 0.24 | 0.32 | 0.25 | 0.41 | 0.37 |
| DSM-5 Level 1 mania | 0.03 | 0.15 | 0.14 | 0.12 | 0.15 | 0.20 |
| DSM-5 Level 1 anxiety | 0.24 | 0.45 | 0.44 | 0.42 | 0.32 | 0.39 |
| PROMIS depression | 0.35 | 0.48 | 0.65 | 0.59 | 0.38 | 0.38 |
| PROMIS anger | 0.27 | 0.29 | 0.35 | 0.29 | 0.52 | 0.42 |
| PROMIS anxiety | 0.27 | 0.55 | 0.52 | 0.48 | 0.38 | 0.48 |

*Note.* To get an idea of the criterion validity of the EMA items (1), weighted Pearson correlations between the aggregated mean of each of the six mood items and retrospective trait-like measure sum scores were conducted. Here, some questionnaire referred to the timeframe before the EMA and some questionnaires referred to a similar timeframe as the EMA allowing to get an idea of the concurrent validity. Abbreviations: ARI = Affective Reactivity Index (2) assessing irritability in the past 7 days; ASRM = Altman Self-Rating Mania Scale (3) assessing manic symptomatology in the past 7 days; DSM-5 Level 1 = DSM-5 Self-Rated Level 1 Cross-Cutting Symptom Measure (4) assessing a broad range of symptoms in the past week; Cross-D = Cross‐cutting Dimensional Scale (5) assessing anxiety in the past 4 weeks; LOT-R = Life-Orientation-Test-Revised (6) assessing optimism; OSLO-3 = Oslo Social Support Scale (7) assessing subjective social support; PHQ-9 = Patient Health Questionnaire module depression (8) assessing depressive symptomatology in the past 2 weeks; PROMIS = Patient-Reported Outcomes Measurement Information System (9) assessing different domains of emotional distress in the past week; PSS-4 = Perceived Stress Scale (10) assessing perceived stress in the past months.

**Table A1.3**

*Partial correlation coefficients of the paths for each group and differences between groups in the contemporaneous networks*

|  |  | Suicidal thought group (ST) | | | |  | Suicidal action group (SA) | | | |  | No suicidal behavior group (No SB) | | | |  | Differences between groups in the r | | |
| --- | --- | --- | --- | --- | --- | --- | --- | --- | --- | --- | --- | --- | --- | --- | --- | --- | --- | --- | --- |
| Variable 1 | Variable 2 | r | SD | p 1<-2 | p 1->2 |  | r | SD | p 1<-2 | p 1->2 |  | r | SD | p 1<-2 | p 1->2 |  | ST vs. SA | ST vs. No SB | SA vs. No SB |
| ANX | STR | 0.11 | 0.01 | .000 | .000 |  | 0.17 | 0.02 | .000 | .000 |  | 0.08 | 0.02 | .000 | .000 |  | 0.06 | 0.03 | 0.09 |
| IRR | STR | 0.19 | 0.02 | .000 | .000 |  | 0.27 | 0.02 | .000 | .000 |  | 0.14 | 0.03 | .000 | .000 |  | 0.08 | 0.05 | 0.13 |
| IRR | ANX | 0.17 | 0.03 | .000 | .000 |  | 0.09 | 0.02 | .029 | .016 |  | 0.17 | 0.04 | .000 | .000 |  | -0.08 | 0.01 | -0.07 |
| HOP | STR | 0.01 | 0.00 | .688 | .912 |  | -0.03 | 0.01 | .437 | .308 |  | 0.04 | 0.01 | .000 | .000 |  | -0.03 | -0.03 | -0.06 |
| HOP | ANX | 0.20 | 0.02 | .000 | .000 |  | 0.27 | 0.04 | .000 | .000 |  | 0.18 | 0.04 | .000 | .000 |  | 0.08 | 0.02 | 0.10 |
| HOP | IRR | 0.08 | 0.01 | .052 | .013 |  | 0.12 | 0.02 | .010 | .004 |  | 0.06 | 0.03 | .000 | .000 |  | 0.04 | 0.02 | 0.06 |
| DEP | STR | 0.02 | 0.00 | .578 | .360 |  | 0.01 | 0.01 | .509 | .668 |  | 0.01 | 0.01 | .153 | .288 |  | -0.01 | 0.01 | 0.00 |
| DEP | ANX | 0.14 | 0.01 | .000 | .000 |  | 0.20 | 0.03 | .000 | .000 |  | 0.15 | 0.04 | .000 | .000 |  | 0.06 | -0.01 | 0.06 |
| DEP | IRR | 0.15 | 0.02 | .000 | .001 |  | 0.17 | 0.01 | .000 | .000 |  | 0.18 | 0.04 | .000 | .000 |  | 0.02 | -0.04 | -0.02 |
| DEP | HOP | 0.45 | 0.03 | .000 | .000 |  | 0.30 | 0.06 | .000 | .000 |  | 0.37 | 0.06 | .000 | .000 |  | -0.16 | 0.08 | -0.08 |
| ANH | STR | 0.16 | 0.02 | .000 | .000 |  | 0.20 | 0.02 | .000 | .000 |  | 0.17 | 0.02 | .000 | .000 |  | 0.04 | -0.01 | 0.03 |
| ANH | ANX | 0.01 | 0.00 | .568 | .848 |  | 0.01 | 0.02 | .801 | .809 |  | 0.01 | 0.02 | .283 | .976 |  | 0.00 | 0.00 | 0.00 |
| ANH | IRR | 0.12 | 0.01 | .000 | .000 |  | 0.08 | 0.00 | .008 | .003 |  | 0.12 | 0.02 | .000 | .000 |  | -0.04 | 0.00 | -0.04 |
| ANH | HOP | 0.03 | 0.00 | .228 | .328 |  | 0.01 | 0.01 | .913 | .770 |  | 0.02 | 0.01 | .034 | .265 |  | -0.02 | 0.01 | -0.01 |
| ANH | DEP | 0.10 | 0.01 | .000 | .000 |  | 0.17 | 0.01 | .000 | .000 |  | 0.11 | 0.02 | .000 | .000 |  | 0.07 | -0.01 | 0.06 |

*Note.* Difference in the edge weight of > 0.07 were found between depression and hopelessness indicating a higher association in the thought (0.45) compared to action (0.30) and no suicidal behavior group (0.37). Further, the action group showed a higher association between irritability and stress as well as hopelessness and anxiety (0.27; 0.27) compared to the thought (0.19; 0.20) and no suicidal behavior group (0.14; 0.18), respectively. In the action group, the association between anxiety and stress was higher compared to the no suicidal behavior group (0.17 vs. 0.08). In the thought group, the association between irritability and anxiety was higher compared to the action group (0.17 vs. 0.09). Abbreviations: ANH: anhedonia, ANX: anxiety, DEP: depression, HOP: hopelessness, IRR: irritability, STR: stress; ST: suicidal thought group; SA: suicidal action group; no SB: no suicidal behavior group; r = partial correlations between mood states; SD = standard deviation; p = p-value in regard to both directions (e.g. p 1<-2 shows the p-value for the directed path between variable two to one).

## Table A1.4

*Fixed effect coefficients of the paths for each of the three groups and differences between the groups in the temporal networks*

|  |  | Suicidal thought group | | |  | Suicidal action group | | |  | No suicidal behavior group | | |  | Differences between groups in the fixed β | | |
| --- | --- | --- | --- | --- | --- | --- | --- | --- | --- | --- | --- | --- | --- | --- | --- | --- |
| from | to | fixed β | SE | p |  | fixed β | SE | p |  | fixed β | SE | p |  | ST vs. SA | ST vs. No SB | SA vs. No SB |
| STR | STR | 0.17 | 0.03 | .000 |  | 0.21 | 0.04 | .000 |  | 0.15 | 0.01 | .000 |  | 0.03 | 0.02 | 0.05 |
| STR | ANX | 0.02 | 0.03 | .420 |  | 0.02 | 0.03 | .519 |  | 0.01 | 0.01 | .157 |  | 0.00 | 0.01 | 0.01 |
| STR | IRR | 0.01 | 0.03 | .593 |  | 0.02 | 0.03 | .437 |  | 0.01 | 0.01 | .272 |  | 0.01 | 0.00 | 0.01 |
| STR | HOP | 0.01 | 0.03 | .592 |  | 0.00 | 0.03 | .996 |  | 0.00 | 0.01 | .906 |  | -0.01 | 0.01 | 0.00 |
| STR | DEP | -0.02 | 0.03 | .477 |  | -0.04 | 0.03 | .268 |  | 0.00 | 0.01 | .849 |  | -0.02 | -0.02 | -0.04 |
| STR | ANH | 0.05 | 0.03 | .100 |  | -0.02 | 0.03 | .536 |  | 0.03 | 0.01 | .000 |  | -0.07 | 0.01 | -0.05 |
| ANX | STR | -0.02 | 0.03 | .491 |  | 0.00 | 0.04 | .939 |  | 0.06 | 0.01 | .000 |  | 0.02 | -0.08 | -0.05 |
| ANX | ANX | 0.10 | 0.04 | .015 |  | 0.15 | 0.05 | .001 |  | 0.11 | 0.02 | .000 |  | 0.06 | -0.02 | 0.04 |
| ANX | IRR | -0.02 | 0.04 | .627 |  | 0.05 | 0.05 | .388 |  | 0.03 | 0.01 | .046 |  | 0.06 | -0.05 | 0.02 |
| ANX | HOP | 0.08 | 0.04 | .028 |  | 0.04 | 0.04 | .308 |  | 0.06 | 0.02 | .000 |  | -0.04 | 0.02 | -0.02 |
| ANX | DEP | 0.01 | 0.03 | .694 |  | 0.15 | 0.05 | .002 |  | 0.03 | 0.02 | .084 |  | 0.14 | -0.01 | 0.13 |
| ANX | ANH | -0.01 | 0.03 | .659 |  | -0.01 | 0.04 | .742 |  | 0.02 | 0.01 | .129 |  | 0.00 | -0.03 | -0.03 |
| IRR | STR | 0.02 | 0.03 | .484 |  | 0.15 | 0.03 | .000 |  | 0.01 | 0.01 | .430 |  | 0.13 | 0.01 | 0.15 |
| IRR | ANX | -0.02 | 0.04 | .577 |  | 0.01 | 0.04 | .811 |  | 0.00 | 0.01 | .739 |  | 0.03 | -0.03 | 0.01 |
| IRR | IRR | 0.15 | 0.04 | .000 |  | 0.23 | 0.05 | .000 |  | 0.10 | 0.01 | .000 |  | 0.09 | 0.04 | 0.13 |
| IRR | HOP | -0.03 | 0.03 | .389 |  | 0.06 | 0.05 | .246 |  | 0.00 | 0.01 | .875 |  | 0.09 | -0.03 | 0.06 |
| IRR | DEP | 0.00 | 0.03 | .948 |  | 0.07 | 0.04 | .082 |  | 0.04 | 0.01 | .004 |  | 0.07 | -0.04 | 0.04 |
| IRR | ANH | -0.03 | 0.03 | .398 |  | 0.09 | 0.03 | .004 |  | -0.02 | 0.01 | .052 |  | 0.12 | -0.01 | 0.11 |
| HOP | STR | 0.04 | 0.04 | .257 |  | 0.00 | 0.04 | .938 |  | 0.02 | 0.01 | .150 |  | -0.04 | 0.02 | -0.02 |
| HOP | ANX | 0.09 | 0.05 | .035 |  | 0.14 | 0.05 | .007 |  | 0.04 | 0.02 | .010 |  | 0.05 | 0.05 | 0.10 |
| HOP | IRR | 0.11 | 0.04 | .008 |  | 0.12 | 0.06 | .036 |  | 0.02 | 0.02 | .250 |  | 0.01 | 0.09 | 0.10 |
| HOP | HOP | 0.09 | 0.05 | .071 |  | 0.12 | 0.06 | .044 |  | 0.09 | 0.02 | .000 |  | 0.04 | 0.00 | 0.03 |
| HOP | DEP | 0.09 | 0.04 | .025 |  | 0.12 | 0.05 | .024 |  | 0.07 | 0.02 | .001 |  | 0.03 | 0.02 | 0.06 |
| HOP | ANH | -0.02 | 0.03 | .441 |  | 0.15 | 0.04 | .000 |  | 0.03 | 0.01 | .020 |  | 0.17 | -0.05 | 0.12 |
| DEP | STR | 0.06 | 0.04 | .130 |  | 0.01 | 0.04 | .763 |  | -0.01 | 0.01 | .501 |  | -0.05 | 0.07 | 0.02 |
| DEP | ANX | 0.04 | 0.04 | .356 |  | 0.04 | 0.03 | .236 |  | 0.04 | 0.01 | .004 |  | 0.00 | 0.00 | 0.00 |
| DEP | IRR | -0.02 | 0.03 | .574 |  | -0.05 | 0.05 | .294 |  | 0.02 | 0.01 | .257 |  | -0.04 | -0.03 | -0.07 |
| DEP | HOP | 0.10 | 0.04 | .011 |  | 0.11 | 0.05 | .019 |  | 0.06 | 0.01 | .000 |  | 0.01 | 0.04 | 0.05 |
| DEP | DEP | 0.18 | 0.04 | .000 |  | 0.09 | 0.05 | .065 |  | 0.11 | 0.02 | .000 |  | -0.10 | 0.07 | -0.03 |
| DEP | ANH | 0.09 | 0.03 | .004 |  | 0.05 | 0.04 | .196 |  | 0.03 | 0.01 | .009 |  | -0.03 | 0.06 | 0.03 |
| ANH | STR | 0.08 | 0.03 | .004 |  | 0.07 | 0.04 | .061 |  | 0.09 | 0.01 | .000 |  | -0.01 | -0.01 | -0.02 |
| ANH | ANX | 0.03 | 0.02 | .198 |  | 0.03 | 0.02 | .238 |  | 0.02 | 0.01 | .009 |  | 0.00 | 0.01 | 0.01 |
| ANH | IRR | 0.01 | 0.03 | .649 |  | 0.04 | 0.04 | .301 |  | 0.04 | 0.01 | .000 |  | 0.03 | -0.02 | 0.01 |
| ANH | HOP | 0.04 | 0.03 | .237 |  | 0.04 | 0.02 | .072 |  | 0.03 | 0.01 | .003 |  | 0.01 | 0.01 | 0.02 |
| ANH | DEP | 0.06 | 0.03 | .027 |  | 0.06 | 0.03 | .046 |  | 0.05 | 0.01 | .000 |  | 0.00 | 0.01 | 0.01 |
| ANH | ANH | 0.21 | 0.04 | .000 |  | 0.21 | 0.04 | .000 |  | 0.22 | 0.01 | .000 |  | 0.01 | -0.01 | 0.00 |

*Note.* Abbreviations: ANH: anhedonia, ANX: anxiety, DEP: depression, HOP: hopelessness, IRR: irritability, STR: stress; ST: suicidal thought group; SA: suicidal action group; no SB: no suicidal behavior group.

## Table A1.5

*Network density indicators for each group and differences between the three groups*

| Network | Suicidal thought | Suicidal  action | No suicidal behavior | Difference  ST vs. SA | Difference  ST vs. no SB | Difference  SA vs. no SB |
| --- | --- | --- | --- | --- | --- | --- |
| Temporal |  |  |  |  |  |  |
| Overall density | 0.115 | 0.142 | 0.067 | 0.027 | 0.048 | 0.075 |
| Internode density | 0.087 | 0.122 | 0.043 | 0.036 | 0.044 | 0.080 |
| Self-loop density | 0.161 | 0.177 | 0.131 | 0.016 | 0.029 | 0.046 |
| Contemporaneous |  |  |  |  |  |  |
| Overall density | 0.178 | 0.185 | 0.146 | 0.007 | 0.032 | 0.039 |

*Note.* Results are based on significant paths. Abbreviations: ST: suicidal thought group; SA: suicidal action group; no SB: no suicidal behavior group.

## Table A1.6

*Network indicators for each mood state and group in the contemporaneous and temporal*

|  |  | Contemporaneous network | | | | | |  | Temporal network | | | | | |
| --- | --- | --- | --- | --- | --- | --- | --- | --- | --- | --- | --- | --- | --- | --- |
|  |  | STR | ANX | IRR | HOP | DEP | ANH |  | STR | ANX | IRR | HOP | DEP | ANH |
| Suicidal thought group |  |  |  |  |  |  |  |  |  |  |  |  |  |  |
| Strenght/Outdegree |  | 0.454 | 0.615 | 0.704 | 0.729 | 0.837 | 0.379 |  | 0.000 | 0.079 | 0.000 | 0.295 | 0.184 | 0.136 |
| Indegree |  |  |  |  |  |  |  |  | 0.078 | 0.094 | 0.111 | 0.177 | 0.148 | 0.085 |
| Suicidal action group |  |  |  |  |  |  |  |  |  |  |  |  |  |  |
| Strenght/Outdegree |  | 0.639 | 0.733 | 0.726 | 0.688 | 0.831 | 0.455 |  | 0.000 | 0.151 | 0.246 | 0.534 | 0.109 | 0.061 |
| Indegree |  |  |  |  |  |  |  |  | 0.154 | 0.140 | 0.122 | 0.109 | 0.334 | 0.242 |
| No suicidal behavior group |  |  |  |  |  |  |  |  |  |  |  |  |  |  |
| Strenght/Outdegree |  | 0.422 | 0.563 | 0.670 | 0.663 | 0.807 | 0.418 |  | 0.034 | 0.140 | 0.037 | 0.136 | 0.121 | 0.215 |
| Indegree |  |  |  |  |  |  |  |  | 0.146 | 0.100 | 0.063 | 0.137 | 0.149 | 0.087 |

*Note.* Indicators based on significant paths. Abbreviations: ANH: anhedonia, ANX: anxiety, DEP: depression, HOP: hopelessness, IRR: irritability, STR: stress.
