# Supplementary material for: Mood dynamics in adolescents and young adults with and without a history of suicidal thoughts and actions: a network approach
Source: BMC Psychiatry. 2026 Jun 11;26:460. doi: 10.1186/s12888-026-08273-w (PMC13262410; doi:10.1186/s12888-026-08273-w)
Supplement: Supplementary file 2 — Supplementary Material 2 [file 12888_2026_8273_MOESM2_ESM.docx]

**Additional File 2**

Voss, C., Kische, H., Ollmann, T. M., Rückert, F., Hoyer, J., Beesdo-Baum, K. Mood Dynamics in Adolescents and Young Adults with and without a History of Suicidal Thoughts and Actions: A Network Approach

**Corresponding author:**

Prof. Katja Beesdo-Baum, PhD

TUD Dresden University of Technology

Institute of Clinical Psychology and Psychotherapy, Behavioral Epidemiology

Chemnitzer Strasse 46

D-01187 Dresden, Germany

Email: katja.beesdo-baum@tu-dresden.de

**Additional File 2 – Description of Additional Analyses: Regression analyses**

## Introduction

Additional analyses were conducted to use a different approach testing group (no suicidal behavior group, suicidal thought group, suicidal action group) as a moderator variable in the associations between the different mood states. Further, covariates could impact the differences in the edges between groups, like the occurrence of a lifetime major depressive disorder and lifetime non-suicidal self-injurious behavior (NSSI). Therefore, these variables were included as covariates in the analyses.

## Method

**Measures**

***History of depressive disorder.*** During the diagnostic interview (DIA-X-5; 1), a wide range of lifetime mental disorders were assessed according to DSM-5 (2). For the present analysis, the following variable was included: any depressive disorder, i.e., major depressive disorder or persistent depressive disorder (dysthymia).

***History of non-suicidal self-injurious behavior (NSSI).*** NSSI was assessed using a dichotomous item of the self-injurious thoughts and behaviors interview for self-harming behavior (SITBI-G; 3, 4) via questionnaire after asking for suicidal behavior: ‘Have you ever purposely hurt yourself without wanting to die?’.

## Statistical Analyses

Multilevel analyses were conducted to test differences edge weights in contemporaneous (< 0.07) and temporal (> 0.09) networks between groups including possible covariates. Multilevel regression analyses were used in order to account for the multilevel structure of the data (assessments nested in subjects). On the one hand, this strategy decreased the risk for false conclusion based on network results due to conditioning on a collider effects (5), on the other hand, this procedure simplifies the analyses by concentrating on one association at the time and therefore might result in other findings. For each edge difference three models were tested: model 1) the interaction between group and the centered [lag (t-1) in temporal associations] predictor variable were entered in the model (the person-mean-centered variable was used as a predictor to capture the pure within-person variations, independent of between-person differences), model 2) the type of day (weekend/week), sex and age variable were added, model 3) the depressive disorder as well as NSSI variable were added. Due to small sample sizes, only the specific predictor/mood state and none of the others were entered in each model.

## Results

***Contemporaneous network.*** Three edge weight differences were tested: irritability and stress, hopelessness and anxiety, hopelessness and anxiety. Results are presented in Figure A2.1 and Table A2.1. No significant group differences were found in the fully adjusted models (model 3).

## Table A2.1

*Contemporaneous network: Results of contemporaneous effects for edges varying between groups*

|  | Model 1 |  |  | Model 2 |  |  | Model 3 |  |  |
| --- | --- | --- | --- | --- | --- | --- | --- | --- | --- |
|  | ß | 95% CI | p | ß | 95% CI | p | ß | 95% CI | p |
| Stress |  |  |  |  |  |  |  |  |  |
| IRR centered | 0.64 | 0.58; 0.69 | <.001 | 0.63 | 0.58; 0.68 | <.001 | 0.63 | 0.58; 0.68 | <.001 |
| ST Group | 0.38 | 0.08; 0.69 | .014 | 0.37 | -0.19; 0.45 | .014 | 0.13 | -0.19; 0.45 | .418 |
| SA Group | 0.40 | 0.05; 0.74 | .024 | 0.32 | -0.40; 0.37 | .067 | -0.02 | -0.40; 0.37 | .938 |
| IRR centered*ST Group | 0.01 | -0.12; 0.13 | .898 | 0.00 | -0.12; 0.13 | .967 | 0.00 | -0.12; 0.13 | .967 |
| IRR centered*SA Group | 0.14 | -0.01; 0.28 | .068 | 0.14 | -0.01; 0.28 | .068 | 0.14 | -0.01; 0.28 | .068 |
| weekend |  |  |  | -0.45 | -0.52;-0.38 | <.001 | -0.45 | -0.52;-0.38 | <.001 |
| female |  |  |  | 0.36 | 0.13; 0.44 | <.001 | 0.29 | 0.13; 0.44 | <.001 |
| age |  |  |  | 0.01 | -0.03; 0.04 | .468 | 0.01 | -0.03; 0.04 | .670 |
| lifetime DD |  |  |  |  |  |  | 0.35 | 0.10; 0.60 | .006 |
| lifetime NSSI |  |  |  |  |  |  | 0.36 | 0.10; 0.62 | .006 |
| Anxiety |  |  |  |  |  |  |  |  |  |
| HOP centered | 0.32 | 0.29; 0.35 | <.001 | 0.32 | 0.29; 0.35 | <.001 | 0.32 | 0.29; 0.35 | <.001 |
| ST Group | 0.22 | 0.09; 0.35 | .001 | 0.22 | 0.09; 0.34 | .001 | 0.08 | -0.06; 0.22 | .266 |
| SA Group | 0.27 | 0.06; 0.48 | .010 | 0.24 | 0.05; 0.44 | .016 | 0.06 | -0.13; 0.25 | .547 |
| HOP centered*ST Group | 0.00 | -0.08; 0.08 | .963 | 0.00 | -0.08; 0.08 | .953 | 0.00 | -0.08; 0.08 | .953 |
| HOP centered*SA Group | 0.02 | -0.07; 0.10 | .716 | 0.02 | -0.07; 0.10 | .723 | 0.02 | -0.07; 0.10 | .723 |
| weekend |  |  |  | -0.03 | -0.05;-0.01 | .009 | -0.03 | -0.05; 0.01 | .008 |
| female |  |  |  | 0.14 | 0.07; 0.21 | <.001 | 0.10 | 0.03; 0.17 | .004 |
| age |  |  |  | -0.01 | -0.02; 0.01 | .303 | -0.01 | -0.03; 0.00 | .153 |
| lifetime DD |  |  |  |  |  |  | 0.20 | 0.05; 0.34 | .007 |
| lifetime NSSI |  |  |  |  |  |  | 0.20 | 0.07; 0.33 | .002 |
| Depression |  |  |  |  |  |  |  |  |  |
| HOP centered | 0.80 | 0.76; 0.84 | <.001 | 0.80 | 0.76; 0.84 | <.001 | 0.80 | 0.76; 0.84 | <.001 |
| ST Group | 0.41 | 0.16; 0.65 | .001 | 0.39 | 0.16; 0.63 | .001 | 0.11 | -0.17; 0.39 | .444 |
| SA Group | 0.62 | 0.27; 0.97 | .001 | 0.55 | 0.21; 0.88 | .001 | 0.16 | -0.18; 0.50 | .358 |
| HOP centered*ST Group | -0.06 | -0.18; 0.06 | .307 | -0.06 | -0.18; 0.06 | .310 | -0.06 | -0.18; 0.06 | .310 |
| HOP centered*SA Group | -0.05 | -0.17; 0.07 | .434 | -0.05 | -0.17; 0.07 | .432 | -0.05 | -0.17; 0.07 | .432 |
| weekend |  |  |  | -0.01 | -0.04; 0.02 | .532 | -0.01 | -0.04; 0.02 | .528 |
| female |  |  |  | 0.33 | 0.22; 0.45 | <.001 | 0.25 | 0.13; 0.36 | <.001 |
| age |  |  |  | 0.00 | -0.03; 0.02 | .876 | -0.01 | -0.03; 0.01 | .472 |
| lifetime DD |  |  |  |  |  |  | 0.42 | 0.17; 0.67 | .001 |
| lifetime NSSI |  |  |  |  |  |  | 0.42 | 0.17; 0.66 | .001 |

*Note.* For each edge difference three models were tested: model 1) the interaction between group and the centered predictor variable were entered in the model (the person-mean-centered variable was used as a predictor to capture the pure within-person variations, independent of between-person differences), model 2) the type of day (weekend/week), sex and age variable were added, model 3) the depressive disorder as well as NSSI variable were added. Abbreviations: HOP: hopelessness, IRR: irritability, ST: suicidal thought group; SA: suicidal action group; DD: depressive disorder; NSSI: non-suicidal self-injurious behavior.

## Figure A2.1

*Results for the interaction effects predictor*group based on varying edge weights in the contemporaneous networks between groups using multi-level modelling*


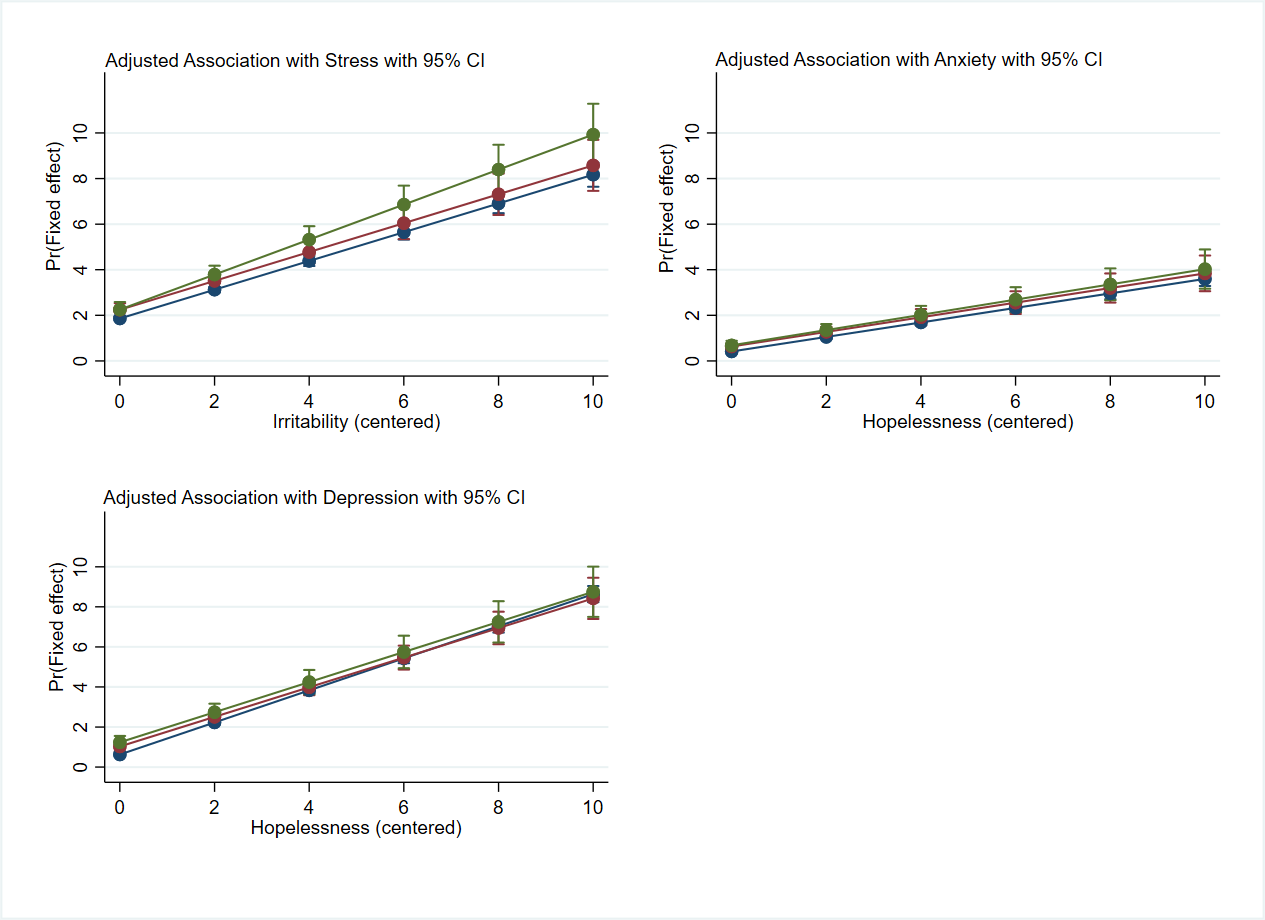


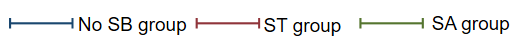


*Note.* Abbreviations: SB: suicidal behavior; ST: suicidal thought; SA: suicidal action.

***Temporal network – internode edges.*** Six internode edge weight differences were tested: anxiety and depression, irritability and stress, irritability and anhedonia, hopelessness and anxiety, hopelessness and irritability, hopelessness and anhedonia. Results are presented in Figure A2.2 and Table A2.2. Main or interaction effects were found for group in model 1 and 2, but only the following interaction effects were found to be significant in model 3: suicidal action group*irritability predicting stress, suicidal action group*irritability predicting anhedonia, suicidal action group*hopelessness predicting anhedonia.

## Table A2.2

*Temporal networks: Results for internode effects for edges varying between groups*

|  | Model 1 |  |  | Model 2 |  |  | Model 3 |  |  |
| --- | --- | --- | --- | --- | --- | --- | --- | --- | --- |
|  | ß | 95% CI | p | ß | 95% CI | p | ß | 95% CI | p |
| Depression |  |  |  |  |  |  |  |  |  |
| ANX (t-1) | 0.32 | 0.25; 0.38 | <.001 | 0.32 | 0.25; 0.38 | <.001 | 0.32 | 0.25; 0.38 | <.001 |
| ST Group | 0.43 | 0.17; 0.69 | .001 | 0.42 | 0.16; 0.67 | .001 | 0.12 | -0.18; 0.43 | .431 |
| SA Group | 0.63 | 0.26; 1.01 | .001 | 0.57 | 0.21; 0.92 | .002 | 0.16 | -0.20; 0.52 | .382 |
| ANX (t-1) * ST Group | -0.08 | -0.22; 0.07 | .315 | -0.08 | -0.22; 0.07 | .310 | -0.08 | -0.22; 0.07 | .312 |
| ANX (t-1) * SA Group | 0.19 | -0.05; 0.42 | .120 | 0.19 | -0.05; 0.42 | .120 | 0.19 | -0.05; 0.42 | .119 |
| weekend |  |  |  | -0.04 | -0.09; 0.01 | .112 | -0.04 | -0.09; 0.01 | .109 |
| female |  |  |  | 0.32 | 0.20; 0.44 | <.001 | 0.23 | 0.11; 0.36 | <.001 |
| age |  |  |  | 0.00 | -0.02; 0.03 | .972 | -0.01 | -0.03; 0.02 | .624 |
| lifetime DD |  |  |  |  |  |  | 0.42 | 0.16; 0.68 | .002 |
| lifetime NSSI |  |  |  |  |  |  | 0.45 | 0.19; 0.72 | .001 |
| Stress |  |  |  |  |  |  |  |  |  |
| IRR (t-1) | 0.21 | 0.15; 0.26 | <.001 | 0.20 | 0.15; 0.26 | <.001 | 0.20 | 0.15; 0.26 | <.001 |
| ST Group | 0.31 | -0.01; 0.63 | .061 | 0.30 | -0.01; 0.61 | .061 | 0.06 | -0.27; 0.39 | .718 |
| SA Group | 0.43 | 0.08; 0.79 | .018 | 0.35 | -0.01; 0.70 | .054 | 0.02 | -0.38; 0.42 | .926 |
| IRR (t-1) * ST Group | 0.07 | -0.08; 0.22 | .353 | 0.06 | -0.08; 0.20 | .397 | 0.06 | -0.08; 0.20 | .395 |
| IRR (t-1) * SA Group | 0.21 | 0.06; 0.37 | .008 | 0.21 | 0.05; 0.38 | .011 | 0.21 | 0.05; 0.38 | .011 |
| weekend |  |  |  | -0.46 | -0.54; -0.38 | <.001 | -0.46 | -0.54; -0.38 | <.001 |
| female |  |  |  | 0.38 | 0.21; 0.55 | <.001 | 0.31 | 0.14; 0.47 | <.001 |
| age |  |  |  | 0.01 | -0.02; 0.05 | .457 | 0.01 | -0.03; 0.04 | .648 |
| lifetime DD |  |  |  |  |  |  | 0.35 | 0.08; 0.62 | .011 |
| lifetime NSSI |  |  |  |  |  |  | 0.35 | 0.08; 0.62 | .012 |
| Anhedonia |  |  |  |  |  |  |  |  |  |
| IRR (t-1) | 0.18 | 0.13; 0.22 | <.001 | 0.17 | 0.12; 0.22 | <.001 | 0.17 | 0.12; 0.22 | <.001 |
| ST Group | 0.42 | 0.00; 0.84 | .048 | 0.42 | -0.01; 0.84 | .053 | 0.03 | -0.43; 0.49 | .897 |
| SA Group | 0.33 | -0.10; 0.75 | .130 | 0.31 | -0.12; 0.74 | .153 | -0.22 | -0.68; 0.24 | .345 |
| IRR (t-1) * ST Group | -0.03 | -0.19; 0.13 | .727 | -0.04 | -0.20; 0.12 | .647 | -0.04 | -0.19; 0.12 | .649 |
| IRR (t-1) * SA Group | 0.22 | 0.08; 0.36 | .002 | 0.22 | 0.08; 0.36 | .002 | 0.22 | 0.08; 0.36 | .002 |
| weekend |  |  |  | -0.52 | -0.62; -0.43 | <.001 | -0.53 | -0.62; -0.43 | <.001 |
| female |  |  |  | -0.05 | -0.26; 0.16 | .647 | -0.17 | -0.38; 0.04 | .120 |
| age |  |  |  | 0.05 | 0.01; 0.10 | .017 | 0.05 | 0.00; 0.09 | .040 |
| lifetime DD |  |  |  |  |  |  | 0.57 | 0.22; 0.91 | .001 |
| lifetime NSSI |  |  |  |  |  |  | 0.58 | 0.25; 0.91 | .001 |
| Anxiety |  |  |  |  |  |  |  |  |  |
| HOP (t-1) | 0.11 | 0.09; 0.14 | <.001 | 0.11 | 0.09; 0.14 | <.001 | 0.11 | 0.09; 0.14 | <.001 |
| ST Group | 0.23 | 0.09; 0.36 | .001 | 0.22 | 0.09; 0.35 | .001 | 0.08 | -0.06; 0.23 | .270 |
| SA Group | 0.26 | 0.04; 0.48 | .019 | 0.24 | 0.03; 0.45 | .028 | 0.04 | -0.16; 0.24 | .677 |
| HOP (t-1) * ST Group | 0.00 | -0.07; 0.06 | .901 | 0.00 | -0.07; 0.06 | .922 | 0.00 | -0.07; 0.06 | .923 |
| HOP (t-1) * SA Group | 0.07 | 0.00; 0.15 | .064 | 0.07 | 0.00; 0.15 | .066 | 0.07 | 0.00; 0.15 | .066 |
| weekend |  |  |  | -0.04 | -0.06; -0.01 | .003 | -0.04 | -0.06; -0.01 | .003 |
| female |  |  |  | 0.13 | 0.06; 0.20 | <.001 | 0.09 | 0.02; 0.16 | .013 |
| age |  |  |  | -0.01 | -0.02; 0.01 | .283 | -0.01 | -0.03; 0.00 | .139 |
| lifetime DD |  |  |  |  |  |  | 0.20 | 0.06; 0.35 | .006 |
| lifetime NSSI |  |  |  |  |  |  | 0.21 | 0.08; 0.35 | .001 |
| Irritability |  |  |  |  |  |  |  |  |  |
| HOP (t-1) | 0.10 | 0.07; 0.13 | <.001 | 0.19 | 0.07; 0.13 | <.001 | 0.10 | 0.07; 0.13 | <.001 |
| ST Group | 0.19 | 0.02; 0.36 | .024 | 0.19 | 0.02; 0.36 | .024 | 0.06 | -0.12; 0.25 | .515 |
| SA Group | 0.25 | 0.06; 0.44 | .011 | 0.23 | 0.04; 0.42 | .016 | 0.05 | -0.16; 0.26 | .638 |
| HOP (t-1) * ST Group | 0.02 | -0.06; 0.11 | .615 | 0.02 | -0.06; 0.11 | .602 | 0.02 | -0.06; 0.11 | .601 |
| HOP (t-1) * SA Group | 0.02 | -0.06; 0.10 | .579 | 0.02 | -0.06; 0.10 | .590 | 0.02 | -0.06; 0.10 | .587 |

## Table A2.2 (continued)

*Temporal networks: Results for internode effects for edges varying between groups*

|  | Model 1 |  |  | Model 2 |  |  | Model 3 |  |  |
| --- | --- | --- | --- | --- | --- | --- | --- | --- | --- |
|  | ß | 95% CI | p | ß | 95% CI | p | ß | 95% CI | p |
| Irritability |  |  |  |  |  |  |  |  |  |
| weekend |  |  |  | -0.03 | -0.07; 0.00 | .071 | -0.03 | -0.07; 0.00 | .070 |
| female |  |  |  | 0.10 | 0.01; 0.18 | .023 | 0.06 | -0.03; 0.14 | .181 |
| age |  |  |  | -0.01 | -0.03; 0.01 | .301 | -0.01 | -0.03; 0.01 | .165 |
| lifetime DD |  |  |  |  |  |  | 0.19 | 0.01; 0.37 | .039 |
| lifetime NSSI |  |  |  |  |  |  | 0.20 | 0.03; 0.37 | .024 |
| Anhedonia |  |  |  |  |  |  |  |  |  |
| HOP (t-1) | 0.21 | 0.16; 0.26 | <.001 | 0.20 | 0.16; 0.25 | <.001 | 0.20 | 0.16; 0.25 | <.001 |
| ST Group | 0.42 | 0.01; 0.84 | .047 | 0.42 | 0.00; 0.84 | .052 | 0.03 | -0.43; 0.49 | .892 |
| SA Group | 0.33 | -0.10; 0.75 | .132 | 0.31 | -0.12; 0.74 | .156 | -0.22 | -0.69; 0.24 | .342 |
| HOP (t-1) * ST Group | -0.09 | -0.21; 0.04 | .181 | -0.07 | -0.20; 0.05 | .243 | -0.07 | -0.20; 0.05 | .244 |
| HOP (t-1) * SA Group | 0.16 | 0.03; 0.30 | .018 | 0.15 | 0.03; 0.28 | .019 | 0.15 | 0.03; 0.28 | .018 |
| weekend |  |  |  | -0.52 | -0.61; -0.43 | <.001 | -0.52 | -0.61; -0.43 | <.001 |
| female |  |  |  | -0.05 | -0.26; 0.17 | .663 | -0.17 | -0.38; 0.05 | .125 |
| age |  |  |  | 0.05 | 0.01; 0.10 | .017 | 0.05 | 0.00; 0.09 | .040 |
| lifetime DD |  |  |  |  |  |  | 0.57 | 0.22; 0.91 | .001 |
| lifetime NSSI |  |  |  |  |  |  | 0.58 | 0.25; 0.92 | .001 |

*Note.* For each edge difference three models were tested: model 1) the interaction between group and the centered lag (t-1) predictor variable were entered in the model (the person-mean-centered variable was used as a predictor to capture the pure within-person variations, independent of between-person differences), model 2) the type of day (weekend/week), sex and age variable were added, model 3) the depressive disorder as well as NSSI variable were added. Abbreviations: ANX: anxiety, HOP: hopelessness, IRR: irritability, ST: suicidal thought group; SA: suicidal action group; DD: depressive disorder; NSSI: non-suicidal self-injurious behavior.

## Figure A2.2

*Results for the interaction effects predictor(t-1)*group based on varying edge weights in the temporal network (internode) between groups using multi-level modelling*


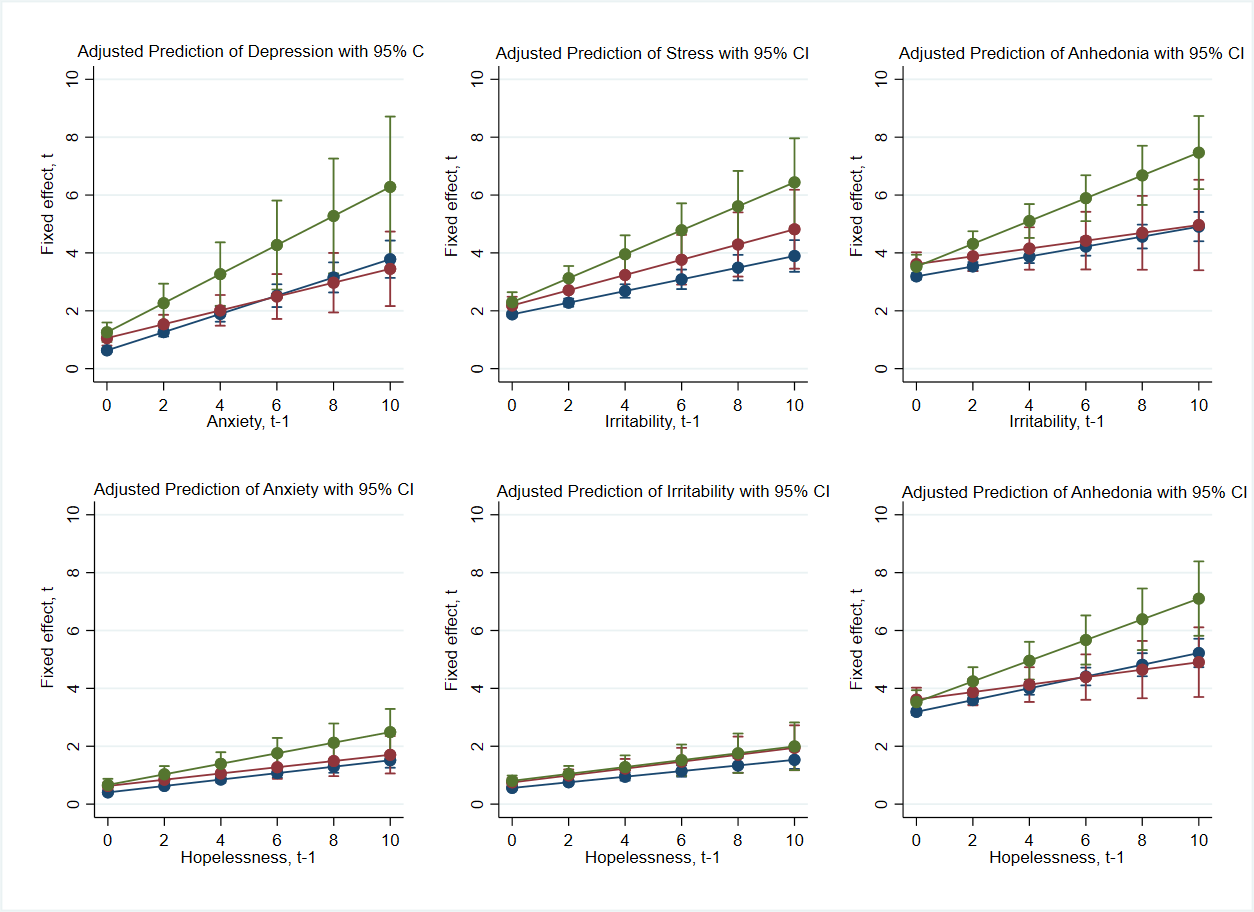


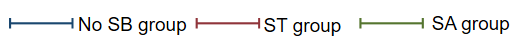


*Note.* Abbreviations: SB: suicidal behavior; ST: suicidal thought; SA: suicidal action.

***Temporal networks - auto-regressive effects.*** Regarding the auto-regressive effects in the temporal networks, two edge weight differences were tested: irritability predicting irritability, depression predicting depression. Results are presented in Figure A2.3 and Table A2.3. Main effects for group were found in model one and two, but not in model 3.

## Table A2.3

*Temporal networks: Results for auto-regressive effects for edges varying between groups*

|  | Model 1 |  |  | Model 2 |  |  | Model 3 |  |  |
| --- | --- | --- | --- | --- | --- | --- | --- | --- | --- |
|  | ß | 95% CI | p | ß | 95% CI | p | ß | 95% CI | p |
| Irritability |  |  |  |  |  |  |  |  |  |
| IRR (t-1) | 0.20 | 0.16; 0.25 | <.001 | 0.20 | 0.16; 0.25 | <.001 | 0.20 | 0.16; 0.25 | <.001 |
| ST Group | 0.19 | 0.02; 0.36 | .027 | 0.19 | 0.02; 0.36 | .027 | 0.06 | -0.13; 0.25 | .531 |
| SA Group | 0.25 | 0.06; 0.44 | .010 | 0.23 | 0.04; 0.42 | .015 | 0.05 | -0.16; 0.26 | .631 |
| IRR (t-1) * ST Group | 0.06 | -0.07; 0.19 | .352 | 0.06 | -0.07; 0.19 | .357 | 0.06 | -0.07; 0.19 | .357 |
| IRR (t-1) * SA Group | 0.10 | -0.04; 0.23 | .150 | 0.10 | -0.04; 0.23 | .151 | 0.10 | -0.04; 0.23 | .151 |
| Weekend |  |  |  | -0.03 | -0.06; 0 | .052 | -0.03 | -0.06; 0.00 | .051 |
| Female |  |  |  | 0.10 | 0.01; 0.18 | .024 | 0.06 | -0.03; 0.14 | .183 |
| Age |  |  |  | -0.01 | -0.03; 0.01 | .308 | -0.01 | -0.03; 0.01 | .168 |
| lifetime DD |  |  |  |  |  |  | 0.19 | 0.01; 0.37 | .038 |
| lifetime NSSI |  |  |  |  |  |  | 0.20 | 0.03; 0.37 | .023 |
|  |  |  |  |  |  |  |  |  |  |
| Depression |  |  |  |  |  |  |  |  |  |
| DEP (t-1) | 0.25 | 0.21; 0.29 | <.001 | 0.25 | 0.21; 0.29 | <.001 | 0.25 | 0.21; 0.29 | <.001 |
| ST Group | 0.43 | 0.17; 0.70 | .001 | 0.42 | 0.17; 0.68 | .001 | 0.13 | -0.17; 0.43 | .404 |
| SA Group | 0.62 | 0.26; 0.99 | .001 | 0.56 | 0.21; 0.91 | .002 | 0.15 | -0.20; 0.50 | .405 |
| DEP (t-1) * ST Group | 0.00 | -0.10; 0.10 | .989 | 0.00 | -0.10; 0.10 | .985 | 0.00 | -0.10; 0.10 | .982 |
| DEP (t-1) * SA Group | 0.10 | -0.03; 0.23 | .120 | 0.10 | -0.03; 0.23 | .120 | 0.10 | -0.03; 0.23 | .120 |
| Weekend |  |  |  | -0.04 | -0.08; 0.00 | .054 | -0.04 | -0.08; 0.00 | .052 |
| Female |  |  |  | 0.33 | 0.21; 0.44 | <.001 | 0.24 | 0.11; 0.36 | <.001 |
| Age |  |  |  | 0.00 | -0.02; 0.02 | .995 | -0.01 | -0.03; 0.02 | .603 |
| lifetime DD |  |  |  |  |  |  | 0.42 | 0.16; 0.68 | .002 |
| lifetime NSSI |  |  |  |  |  |  | 0.45 | 0.19; 0.71 | .001 |

*Note.* For each edge difference three models were tested: model 1) the interaction between group and the centered lag (t-1) predictor variable were entered in the model (the person-mean-centered variable was used as a predictor to capture the pure within-person variations, independent of between-person differences), model 2) the type of day (weekend/week), sex and age variable were added, model 3) the depressive disorder as well as NSSI variable were added. Abbreviations: DEP: depression, IRR: irritability, ST: suicidal thought group; SA: suicidal action group; DD: depressive disorder; NSSI: non-suicidal self-injurious behavior.

## Figure A2.3

*Results for the interaction effects predictor(t-1)*group based on varying edge weights in the temporal network (auto-regressive effect) between groups using multi-level modelling*


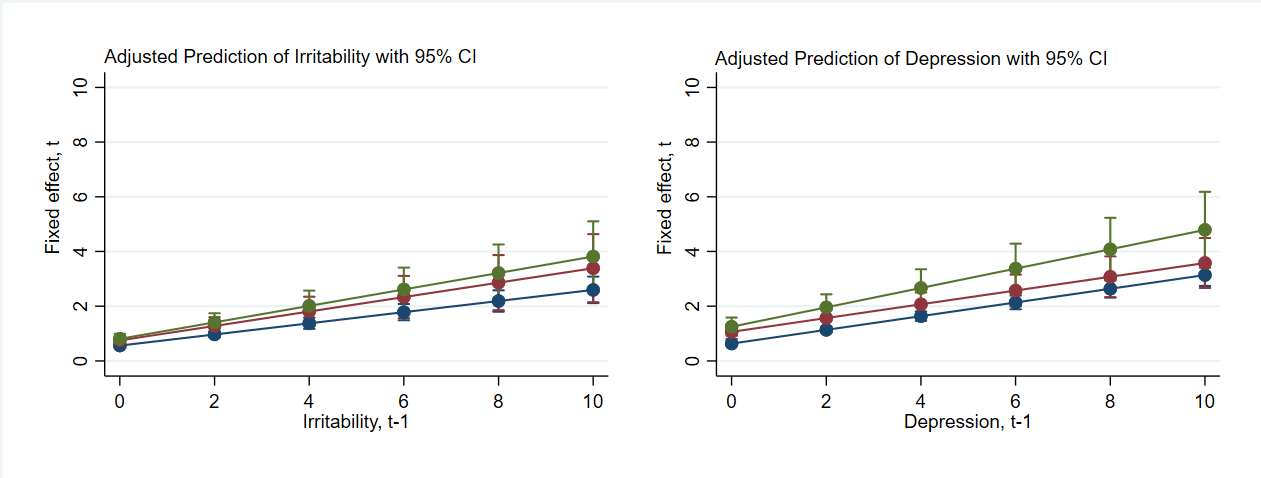


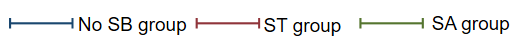


*Note.* Abbreviations: SB: suicidal behavior; ST: suicidal thought; SA: suicidal action.

# References

1. Hoyer J, Voss C, Strehle J, Venz J, Pieper L, Wittchen HU, et al. Test-retest reliability of the computer-assisted DIA-X-5 interview for mental disorders. BMC Psychiatry. 2020;20(1):280.

2. American Psychiatric Association (APA). Diagnostic and Statistical Manual of Mental Disorders, Fifth Edition (DSM-5). 5th ed. Arlington, VA: American Psychiatric Association; 2013.

3. Nock MK, Holmberg EB, Photos VI, Michel BD. Self-Injurious Thoughts and Behaviors Interview: development, reliability, and validity in an adolescent sample. Psychol Assess. 2007;19(3):309-17.

4. Fischer G, Ameis N, Parzer P, Plener PL, Groschwitz R, Vonderlin E, et al. The German version of the self-injurious thoughts and behaviors interview (SITBI-G): a tool to assess non-suicidal self-injury and suicidal behavior disorder. BMC Psychiatry. 2014;14:265.

5. de Ron J, Fried EI, Epskamp S. Psychological networks in clinical populations: investigating the consequences of Berkson's bias. Psychol Med. 2021;51(1):168-76.
