# Supplementary material for: Mood dynamics in adolescents and young adults with and without a history of suicidal thoughts and actions: a network approach
Source: BMC Psychiatry. 2026 Jun 11;26:460. doi: 10.1186/s12888-026-08273-w (PMC13262410; doi:10.1186/s12888-026-08273-w)
Supplement: Supplementary file 3 — Supplementary Material 3 [file 12888_2026_8273_MOESM3_ESM.docx]

**Additional File 3**

Voss, C., Kische, H., Ollmann, T. M., Rückert, F., Hoyer, J., Beesdo-Baum, K. Mood Dynamics in Adolescents and Young Adults with and without a History of Suicidal Thoughts and Actions: A Network Approach

**Corresponding author:**

Prof. Katja Beesdo-Baum, PhD

TUD Dresden University of Technology

Institute of Clinical Psychology and Psychotherapy, Behavioral Epidemiology

Chemnitzer Strasse 46

D-01187 Dresden, Germany

Email: katja.beesdo-baum@tu-dresden.de

**Additional File 3 – Description of Additional Analyses: Permutation tests**

## Introduction

The comparison of networks of different groups was mostly based on visually comparing the parameter estimates in previous research (1). A recent study published two approaches, which could be used to get inferences in the comparison of two groups. Here, the mlVAR R-package (2) and the mnet R-package (3) were used to conduct a parametric test and a nonparametric permutation test (1). Using simulation studies, Haslbeck and colleagues (1) recommended using the nonparametric permutation test with Np = 1000 perturbations. There are several pros and cons, which are explained in detail in their publication. The largest disadvantage of the nonparametric permutation test is the high runtime, making it not feasible when comparing large data sets. Our goal was to compare the three groups to underpin our understanding of the Ideation-to-Action pathway.

## Method

In the present additional analyses, we compared the suicidal thought only group with the suicidal action group, the no suicidal behavior group with the suicidal though only, and the no suicidal behavior group with the suicidal action group using the nonparametric permutation test with Np = 1000 and set the number of cores to 12 following the Tutorial and provided R-code by Haslbeck and colleagues (1).

## Results

***Suicidal though only group vs. suicidal action group.*** The runtime was 5.52 min on 32cores. The results can be found in Figure A3.1 (a and b) for the fixed temporal effects. The temporal associations from one moment to the next were stronger in the suicidal action compared to the suicidal thought group for the following edges: irritability predicting stress (p = .014), irritability predicting anhedonia (p = .013), hopelessness predicting anhedonia (p = .009), and anxiety predicting depression (p = .048).

***No suicidal behavior group vs. suicidal though only group.*** The runtime was 222.21 min on 32 cores. The results can be found in Figure A3.2 (c and d) for fixed temporal effects. The temporal associations from one moment to the next were stronger in the suicidal thought compared to the no suicidal behavior group for the following edges: anxiety predicting stress (p = .046).

***No suicidal behavior group vs. suicidal action group.*** The runtime was 2796.399 min on 32 cores. The results can be found in Figure A3.3 (e and f) for the fixed temporal effects. The temporal associations from one moment to the next were stronger in the suicidal action compared to the no suicidal behavior group for the following edges: irritability predicting irritability (p = .017), irritability predicting stress (p < .001), irritability predicting anhedonia (p = .001), hopelessness predicting anhedonia (p = .003), and anxiety predicting depression (p = .033).

## Figure A3.1

*Results of the nonparametric permutation test comparing the fixed temporal effects of the suicidal thought and suicidal action group.*

a) Differences between ST and SA group b) Significant differences between ST and SA group

c) Differences between no SB and ST group d) Significant differences between no SB and ST group

e) Differences between no SB and SA group f) Significant differences between no SB and SA group

*Note.* Blue edges indicate positive associations, red edges indicate negative associations. On the left side, all differences are plotted, while on the right side only the significant differences with a = 0.05 are plotted. Abbreviations: no SB: no suicidal behavior; ST: suicidal thought; SA: suicidal action, ANH: anhedonia, ANX: anxiety, DEP: depression, HOP: hopelessness, IRR: irritability, STR: stress.

# References

1. Haslbeck JMB, Epskamp S, Waldorp LJ. Testing for group differences in multilevel vector autoregressive models. Behav Res Methods. 2025;57(3):100.

2. Epskamp S, Waldorp LJ, Mottus R, Borsboom D. The Gaussian Graphical Model in Cross-Sectional and Time-Series Data. Multivariate Behav Res. 2018;53(4):453-80.

3. Haslbeck J. Mnet: Modeling group differences and moderation effects in statistical network models. R package version 0.1.4. <https://cran.r-project.org/web/packages/mnet/mnet.pdf>. 2025.
